# The efficacy and safety of regorafenib/fruquintinib combined with PD-1/PD-L1 for metastatic colorectal cancer: a meta-analysis based on single-arm studies

**DOI:** 10.3389/fimmu.2025.1579293

**Published:** 2025-05-29

**Authors:** Fan Yang, Ying Mao, Hanyu Huang, Wen Luo, Li Liu, Wenzhi Chen

**Affiliations:** ^1^ State Key Laboratory of Ultrasound in Medicine and Engineering, College of Biomedical Engineering, Chongqing Medical University, Chongqing, China; ^2^ Department of Oncology, Suining Central Hospital, Suining, China

**Keywords:** metastatic colorectal cancer, regorafenib, fruquintinib, PD-1/PD-L1 inhibitors, meta-analysis

## Abstract

**Objective:**

The efficacy of regorafenib or fruquintinib in combination with PD-1/PD-L1 inhibitors for metastatic colorectal cancer (mCRC) treatment has not been elucidated. This study aims to systematically evaluate the efficacy and safety of this combination therapy.

**Methods:**

PubMed, Embase, Cochrane Library, and Web of Science were systematically retrieved until July 24, 2024. A meta-analysis was carried out for the overall objective response rate (ORR), disease control rate (DCR), progression-free survival (PFS), overall survival (OS), and the incidence of grade 3 or higher treatment-related adverse events (AEs). Non-overlapping 95% confidence intervals (CIs) were considered statistically significant.

**Results:**

26 studies encompassing 1,409 patients were analyzed. Pooled analysis revealed an ORR of 6% (95% CI: 3%-12%), a DCR of 62% (95% CI: 55%-68%), a median PFS of 3.84 months (95% CI: 3.19-4.49 months), a median OS of 13.08 months (95% CI: 10.17-16.00 months), and an incidence rate of grade 3–4 AEs of 21% (95% CI: 15%-28%). In subgroup analyses, the fruquintinib-based regimen demonstrated significantly superior efficacy compared to regorafenib-based therapy, with higher ORR (16% [95% CI: 13%-21%] vs 3% [95% CI: 1%-9%]), DCR (79% [95% CI: 72%-85%] vs 54% [95% CI: 47%-61%]), and median PFS (5.40 months [95% CI: 4.60-6.19] vs 3.00 months [95% CI: 2.47-3.52]). Median OS was numerically but not significantly longer with fruquintinib (14.35 months [95% CI: 10.68-18.02] vs 12.70 months [95% CI: 8.79-16.61]). Liver metastasis status strongly influenced outcomes, with significantly lower ORR (3% [95% CI: 1%-13%] vs 49% [95% CI: 32%-76%]) and shorter median PFS (2.37 months [95% CI: 1.77-2.96] vs 3.50 months [95% CI: 3.09-3.91]) in patients with liver involvement.

**Conclusion:**

The combination of regorafenib or fruquintinib with PD-1/PD-L1 shows moderate efficacy and acceptable safety in the treatment of mCRC. The fruquintinib-based regimen may be superior to the regorafenib-based regimen, and patients without liver metastasis may derive greater benefits. These findings offer new insights for treating mCRC, although they should be validated through large randomized controlled trials.

**Systematic review registration:**

https://www.crd.york.ac.uk/PROSPERO, identifier CRD42024582268

## Introduction

Colorectal cancer (CRC), one of the most prevalent cancers worldwide, represents the second largest cause of cancer-related death after lung cancer (1). Despite significant advances in early screening techniques for cancer over the past decade, more than 20% of CRC patients have already developed metastasis at the time of diagnosis, which presents a substantial challenge for treatment (2). Regarding refractory metastatic colorectal cancer (mCRC) that has progressed after standard second-line treatment, current therapeutic methods are limited. Regorafenib, fruquintinib, and trifluridine have been approved as standard third-line treatment for patients with mCRC, but their efficacy remains suboptimal, with the objective response rate (ORR) of 1% - 5% and median overall survival(OS) of typically fewer than ten months (3–7). Therefore, it is urgent to develop novel therapeutic approaches for better survival.

In recent years, significant breakthroughs have been achieved in immune checkpoint inhibitors (ICIs) in the field of oncology (8). However, for microsatellite stable (MSS) patients, who constitute 95% of mCRC cases, the efficacy of monotherapy with ICIs is extremely limited (8, 9). Research has demonstrated that anti-angiogenic agents boost the efficacy of immunotherapy owing to multiple mechanisms. On the one hand, they can reverse T-cell dysfunction in the tumor microenvironment by inhibiting the VEGF/VEGFR signaling pathway. On the other hand, they can promote anti-tumor immune responses by modulating immune cell recruitment and activation (10–12). Regorafenib and fruquintinib, selective multi-target tyrosine kinase inhibitors, mainly suppress tumor angiogenesis by disrupting the vascular endothelial growth factor receptor (VEGFR) signaling pathway. Both agents have been widely adopted as standard third-line therapies for mCRC patients (4, 5). Currently, regorafenib or fruquintinib in combination with ICIs has been shown to reverse immune suppression and demonstrate promising efficacy in MSS or mismatch repair proficient (pMMR) mCRC (13–15). In the Phase Ib REGONIVO trial, regorafenib combined with nivolumab exhibited encouraging antitumor activity in mCRC sufferers (14). However, as most of the existing studies are small, single-center investigations with heterogeneous results, the overall efficacy and safety of this combination therapy still require further evaluation. Moreover, the differential responses to treatment among various patient subgroups (e.g., those with or without liver metastasis) and the factors influencing these responses have not been systematically investigated.

Therefore, our meta-analysis systematically shed light on the efficacy and safety of regorafenib/fruquintinib combined with PD-1/PD-L1 inhibitors in the mCRC cohort. After integrating available clinical research data, this study seeks to offer key evidence for the clinical treatment of mCRC.

## Materials and methods

The protocol for this meta-analysis was pre-registered in PROSPERO (No: CRD42024582268). The entire research strictly followed the Preferred Reporting Items for Systematic Reviews and Meta-Analyses (PRISMA) (16) guidelines to ensure the transparency, integrity, and reproducibility of the study. The PRISMA checklist is provided in the **Supplementary Materials**.

### Search strategy

PubMed, Embase, Cochrane Library, and Web of Science databases were systematically searched. The most recent search was conducted on July 24, 2024. The search terms included: “metastatic colorectal cancer”, “Regorafenib”, “Fruquintinib” and “Immune Checkpoint Inhibitors”. Only English articles were retrieved. Additionally, the references of eligible ones were checked to identify relevant studies. The strategy is detailed in **Supplementary Table S1**.

### Eligibility criteria

Studies that met the following inclusion criteria were eligible for inclusion in this meta-analysis: 1) Population: Patients diagnosed with mCRC; 2) Intervention: Patients receiving regorafenib/fruquintinib in combination with PD-1/PD-L1 inhibitors; 3) Study Type: This study included prospective clinical trials (Phases I, II, and III), retrospective cohort studies, and real-world evidence studies; 4) Outcomes: Studies reporting clinical oncological outcomes, including ORR, disease control rate (DCR),progression-free survival (PFS), OS, and adverse events (AEs).

The following studies were excluded: 1) animal studies, reviews, meta-analyses, duplicate studies, case reports, conference abstracts, or editorials; 2) studies from which relevant data could not be extracted for the primary research objectives; 3) non-English publications.

The studies were screened by two independent, trained researchers (FY and YM) via EndNote (Clarivate Analytics, Philadelphia, PA, USA) strictly following the predefined inclusion and exclusion criteria. The screening process was divided into two stages: an initial screening of the titles and abstracts, and a full-text review of the remaining articles. A third senior researcher (LL) was consulted to settle disagreements and reach agreements.

### Data extraction and quality assessment

Two researchers independently extracted the necessary information and appraised the quality of the included studies. They extracted author, publication year, country, sample size, age, design, Eastern Cooperative Oncology Group performance status (ECOG PS), previous lines of treatment, intervention drugs, initial drug dose, categories of ICIs, metastatic sites, KRAS/NRAS/BRAF mutations, potential biomarkers, median follow-up length, as well as clinical and safety outcomes such as ORR, DCR, OS, PFS, and grade 3 or higher AEs or immune-related adverse events (irAEs).

The modified Methodological Index for Non-Randomized Studies (MINORS) score tool was utilized for evaluating the quality of the eligible studies. Eight items make up the MINORS score, which was from 0 to 16. Each item was rated on a 3-point scale (0–2). A score below 8 signified low quality, 9–12 denoted moderate quality, and 13–16 indicated high quality.

### Statistical analysis

Data were analyzed using STATA 15.1 software (StataCorp LP, College Station, TX, USA) and R 4.4.1. Heterogeneity was assessed through Q-test and I² statistic. A p-value <0.1 was considered indicative of significant statistical heterogeneity. If significant heterogeneity was identified (p <0.1 and I² > 50%), a random-effects model was adopted; otherwise, a fixed-effects model was employed. Potential sources of heterogeneity were explored through subgroup analyses primarily based on liver metastasis, treatment regimen (PD-1/PD-L1 + regorafenib vs PD-1/PD-L1 + fruquintinib), and country. A lack of overlap in the 95% confidence intervals (CIs) was deemed indicative of statistical significance (17). Additionally, the stability and reliability of pooled results were evaluated through sensitivity analyses. Lastly, publication bias was detected via Begg’s rank correlation and Egger’s regression tests, with a p-value <0.05 considered statistically significant. Any publication bias was corrected through the trim-and-fill method. Except for heterogeneity tests, all statistical tests were two-sided and p <0.05 signified statistically significant.

## Results

### Study selection

**Figure 1:**
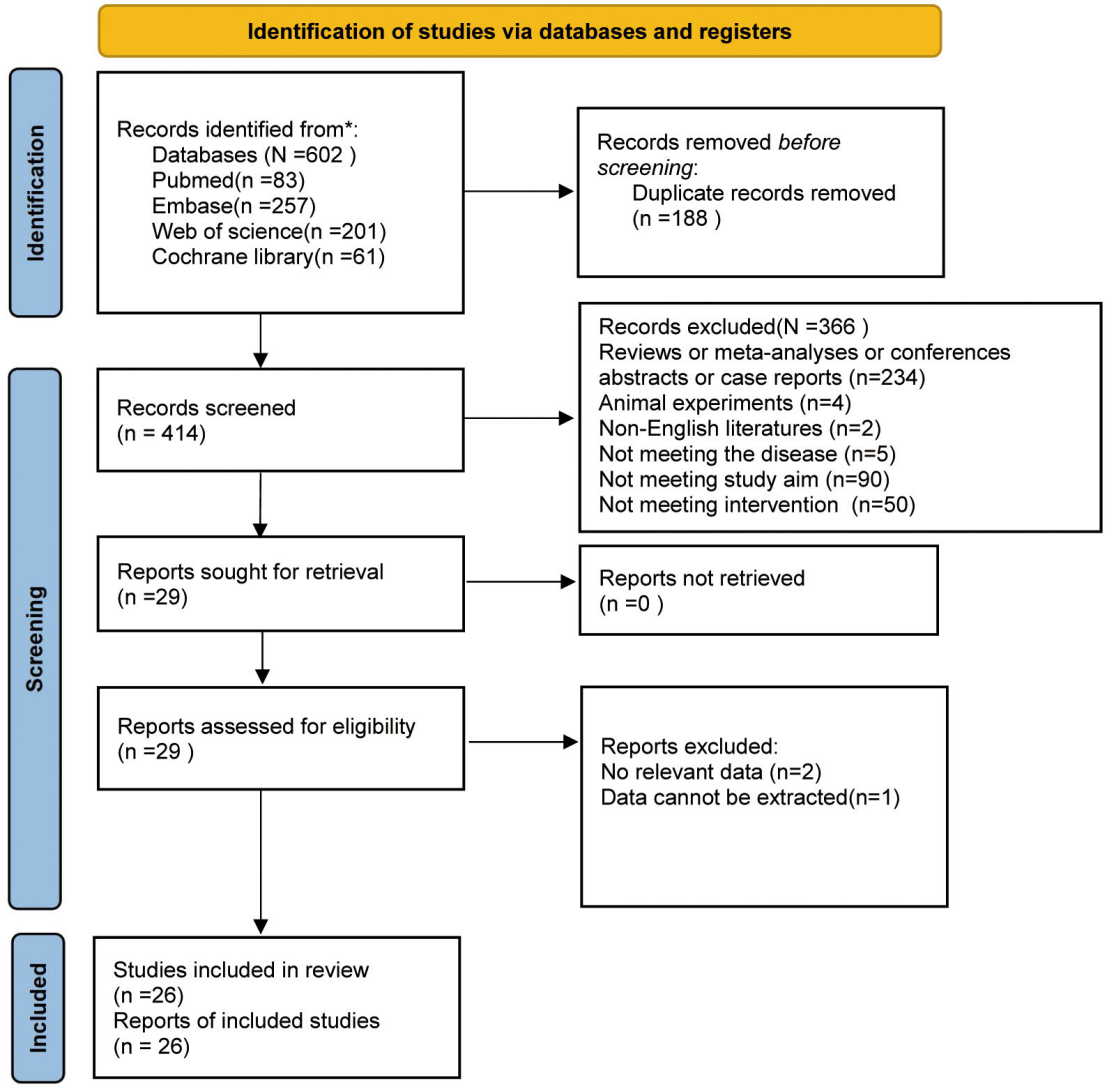
Flow diagram of meta-analysis for study inclusion/exclusion.

Initially, 602 studies were retrieved from PubMed (n=83), Embase (n=257), Web of Science (n=201), and Cochrane Library (n=61). After duplicates were removed, 414 studies were left. Following title and abstract screening, 29 were retained. After a careful full-text review of the remaining articles, 3 were ostracized because of missing data or data availability. A flowchart of the study selection process is illustrated in **Figure 1**.

### Baseline characteristics

26 studies encompassing 1,409 patients were selected. They were conducted in several countries, including China, France, Australia, the United States, and Japan. The average age ranged from 51.54 ± 11.11 to 68.29 ± 4.23 years, with the proportion of male participants ranging from 37.5% to 88.9%. 93% of patients exhibited an ECOG PS of 0-1, and 98% had received at least two prior lines of therapy. The reported incidence of KRAS/NRAS mutations ranged from 17.4% to 71.2%. Among the included studies, 9 (34.6%) were prospective clinical trials, while the rest comprised retrospective analyses or real-world evidence studies. The proportion of patients with liver metastases was 61.4%. Of the included studies, 25 utilized PD-1 inhibitors(sintilimab, carrelizumab, toripalimab, tislelizumab, nivolumab, pembrolizumab, nofazinlimab), while one study selected a PD-L1 inhibitor(avelumab). Information on each study is detailed in **Table 1** and **Supplementary Table S2**.

**Table 1:** Characteristics of the studies included in the meta-analysis.

Study, year	Nation	Study design		Intervention	Sample size	Mean age, years	Median follow-up, months	Liver metastasis		MSI status		PD-1/PD-L1 inhibitors	Quality score
									pMMR/ MSS	dMMR/ MSI-H	Unknown		
Chen et al., 2022 (39)	China	Retrospective study		Regorafenib	24	68.29±4.23	16.2	15(62.5%)	19(79.2%)	1(4.2%)	4(16.7%)	PD-1 inhibitors	10
Cousin et al., 2021 (27)	France	Prospective study, Phase II		Regorafenib	47	60.63±12.84	7.2	35(74.5%)	47(100%)	0	0	PD-L1 inhibitors	14
Wang et al., 2023 (40)	China	Retrospective study		Regorafenib	53	<60,28((53%); ≥60,25(47%)	11.3	41(77.4%)	39 (76.6%)	4 (7.5%%)	10 (18.9%)	PD-1 inhibitors	10
Zhang et al., 2022 (30)	China	Retrospective study		Fruquintinib	110	<65, 9182.7%); ≥65,19 (17.3%)	NR	60(54.5%)	110(100%)	0	0	PD-1 inhibitors	10
An et al., 2024 (41)	China	Retrospective cohort study		Regorafenib	81	53.74±12.79	18.8	50 (61.7%)	81(100%)	0	0	PD-1 inhibitors	10
				Fruquintinib	95	53.71±11.89	18.8	53 (55.8%)	95(100%)	0	0	PD-1 inhibitors	10
Dai et al., 2023 (29)	China	Real-world study		Regorafenib	21	52.14±10.85	NR	14 (66.7%)	21(100%)	0	0	PD-1 inhibitors	14
Day et al., 2023 (42)	Australia	Prospective study, Phase IIa		Regorafenib	14	50.6±9.66	6.7	NR	10 (71.4%)	0	4 (28.6%)	PD-1 inhibitors	14
Fakih et al., 2023 (43)	USA	Prospective study, Phase II		Regorafenib	70	57.14±3.38	NR	47 (67.1%)	70(100%)	0	0	PD-1 inhibitors	16
Fukuoka et al., 2020 (14)	Japan	Prospective study, Phase Ib		Regorafenib	25	54.74±11.71	NR	13 (52%)	24 (96%)	1 (4%)	0	PD-1 inhibitors	12
Gou et al., 2022 (44)	China	Real-world study		Fruquintinib	45	54.56±12.71	NR	36(80%)	45(100%)	0	0	PD-1 inhibitors	15
Guo et al., 2023 (45)	China	Prospective study, Phase 1b/2		Fruquintinib	44	55.58±10.23	NR	NR	25(56.8%)	0	19(43.2%)	PD-1 inhibitors	10
Jiang et al., 2021 (15)	China	Retrospective study		Regorafenib/ Fruquintinib	16	53.17±11.59	NR	11 (68.8%)	16(100%)	0	0	PD-1 inhibitors	15
Kim et al., 2022 (28)	USA	Prospective study, Phase I/Ib		Regorafenib	52	55.83±10.63	NR	38 (73.1%)	52(100%)	0	0	PD-1 inhibitors	10
Li et al., 2020 (46)	China	Retrospective study		Regorafenib	23	50.83±10.37	7.9	13 (56.5%)	23(100%)	0	0	PD-1 inhibitors	10
Li et al., 2023 (47)	China	Real-world study		Fruquintinib	47	58.36±7.43	10.791	26 (55.3%)	NR	NR	NR	PD-1 inhibitors	10
Li et al., 2022 (48)	China	Retrospective study		Regorafenib	103	55.28±11.76	5.3	59 (57.3%)	103 (100.0%)	0	0	PD-1 inhibitors	13
Ma et al., 2023 (49)	China	Prospective study, Phase II		Fruquintinib	19	51.54±11.11	NR	13( 68.42%)	19(100.0%)	0	0	PD-1 inhibitors	13
Nie et al., 2022 (50)	China	Real-world study		Regorafenib	42	58.12±8.96	NR	24 (57.1%)	42 (100%)	0	0	PD-1 inhibitors	13
				Fruquintinib	30	55.76±11.27	NR	20 (66.7%)	30 (100%)	0	0	PD-1 inhibitors	11
Qu et al.,2024 (51)	China	Retrospective study		Regorafenib	161	58.1±11.3	28.4	80 (49.7%)	79 (49.1%)	0	82 50.9%)	PD-1 inhibitors	11
Sun et al., 2021 (52)	China	Retrospective study		Fruquintinib	28	54.6 ± 11.7	NR	18 (64.3%)	28(100.0%)	0	0	PD-1 inhibitors	15
				Regorafenib	23	53.0 ± 12.02	NR	20 (87.0%)	23(100.0%)	0	0	PD-1 inhibitors	11
Wang et al., 2020 (53)	USA	Retrospective study		Regorafenib	18	60.31±9.88	7	14 (77.8%)	18(100.0%)	0	0	PD-1 inhibitors	11
Wang et al., 2021 (54)	China	Prospective study, Phase Ib/II		Regorafenib	42	53±7.45	NR	30 (71.4%)	42 (100.0%)	0	0	PD-1 inhibitors	15
Xu et al., 2022 (55)	China	Retrospective study		Regorafenib	30	≥60,12 (40%); <60,18 (60.0%)	12	18 (60.6%)	30 (100.0%)	0	0	PD-1 inhibitors	15
Yang et al., 2022 (56)	China	Retrospective study		Regorafenib	84	62.37±9.44	5.5	55 (65%)	76 (90%)	0	8 (9.5%)	PD-1 inhibitors	10
Yu et al.,2021 (57)	China	Retrospective study		Regorafenib	33	53.64±10.34	NR	20(60.6%)	NR	NR	NR	NR	14
Fakih et al.,2023 (58)	USA	Prospective study, Phase I		Regorafenib	29	55.12±9.62	NR	7(21.1%)	29(100.0%)	0	0	PD-1 inhibitors	10
Study,year		ECOG PS				Previous lines of treatment			KRAS/ NRAS mutation		BRAF mutation		Peritoneum metastasis
		0	1		2	≤1	2	≥3					
Chen et al., 2022 (39)		5 (20.8%)	16 (66.7%)		3 (12.5%)	0	13(54.2%)	11(45.8%)	16 (66.7%)		1(4.2%)		4 (16.7%)
Cousin et al., 2021 (27)		28 (60%)	19 (40%)		0	6(12.8%)	14 (29.8%)	27 (57.4%)	30(64%)		3 (6%)		15 (32%)
Wang et al., 2023 (40)		NR	NR		NR	0	53(100%)		32(60.4%)		1 (2%)		12 (23%)
Zhang et al., 2022 (30)		31 (28.2%)	67 (60.9%)		12 (10.9%)	0	32 (29.1%)	78 (70.9%)	54 (49.1%)		6 (5.4%)		25 (22.7%)
An et al., 2024 (41)		77 (95.1%)			4 (4.9%)	44 (54.3%)		37(45.7%)	37(45.7%)		8 (9.9%)		NR
		85 (89.5%)			10 (10.5%)	64 (67.4%)		31 (32.6%)	42 (44.2%)		7 (7.4%)		NR
Dai et al., 2023 (29)		6 (28.6%)	15 (71.4%)		0	0	0	21(100%)	8(38.1%)		3 (14.3%)		6 (28.6%)
Day et al., 2023 (42)		9 (64.3%)	5 (35.7%)		0	4.0 (2, 7)			7 (50%)		1 (7.1%)		NR
Fakih et al., 2023 (43)		36 (51%)	34 (49%)		0	3(4.3%)	30 (42.9%)	37(52.9%)	43 (61%)		3 (4%)		6 (9%)
Fukuoka et al., 2020 (14)		25 (100%)	0		0	0	5 (20%)	20 (80%)	6 (24%)		NR		4 (16%)
Gou et al., 2022 (44)		33(73.3%)			12(26.7%)	NR	NR	NR	24( 53.3%)		NR		NR
Guo et al., 2023 (45)		17 (38.6%)	27 (61.4%)		0	1 (2.3%)	30 (68.2%)	13 (29.5%)	NR		NR		NR
Jiang et al., 2021 (15)		6 (37.5%)	10 (62.5%)		0	0	7 (43.7%)	9(56.3%)	NR		NR		5 (31.3%)
Kim et al., 2022 (28)		20 (38.5%)	32 (61.5%)		0	0	30 (57.7%)	22(42.3%)	37 (71.2%)		NR		11 (39.3%)
Li et al., 2020 (46)		6 (26.1%)	14 (60.9%)		3 (13.0%)	0	8 (34.8%)	15 (65.2%)	12 (52.2%)		1 (4.3%)		NR
Li et al., 2023 (47)		41 (87.2%)			6 (12.8%)	26 (55.3%)		21 (44.7%)	24(51.1%)		4 (8.9%)		NR
Li et al., 2022 (48)		36 (35.0%)	61 (59.2%)		6 (5.8%)	0	58 (56.3%)	45(43.7%)	40 (38.8%)		6 (5.8%)		14 (13.5%)
Ma et al., 2023 (49)		8 (42.1%)	11(57.9%)		0	0	19(100%)		8 (50.00%)		0 (0.00)		1 (5.26%)
Nie et al., 2022 (50)		33 (78.6%)			9 (21.4%)	0	0	42(100%)	20(47.6%)		1 (2.4%)		9 (21.4%)
		25 (83.3%)			5 (16.7%)	0	0	30(100%)	13(43.3%)		0 (0.00)		11 (36.7%)
Qu et al.,2024 (51)		157 (97.5%)			4 (2.5%)	0	161(100%)		69(58.4%)		8 (11.0%)		NR
Sun et al., 2021 (52)		13 (46.4%)	11 (39.3%)		4 (14.3%)	0	0	28(100%)	10 (35.7%)		0 (0.0)		7 (25.0%)
		8 (34.8%)	11 (47.8%)		4 (17.4%)	0	0	23(100%)	4 (17.4%)		1 (4.3%)		6 (26.1%)
Wang et al., 2020 (53)		5 (27.8%)	13 (72.2%)		0	NR	NR	NR	NR		NR		NR
Wang et al., 2021 (54)		3 (7.1%)	39(92.9%)		0	0	27(64.3%)	15(35.7%)	21 (50%)		2 (4.8%)		10 (23.8%)
Xu et al., 2022 (55)		21(70.0%)			9 (30.0%)	0	0	30(100%)	7 (23.3%)		0 (0.00)		6 (20.0%)
Yang et al., 2022 (56)		21 (25.0%)	61 (72.6%)		2 (2.4%)	8(9.5%)	25 (29.8%)	51(60.7%)	45 (54%)		3 (4%)		18 (21%)
Yu et al.,2021 (57)		10(30.3%)	23(69.7%)		0	0	16(48.5%)	17(51.5%)	8(24.2%)		NR		NR
Fakih et al.,2023 (58)		17(58.6%)	12(41.4%)		0	1 (3.4%)	14 (48.3%)	14 (48.3%)	20 (69.0%)		3 (10.3%)		NR

NR, not reported; ORR, overall response rate; DCR, disease control rate; OS, overall survival; PFS, progression-free survival; AEs, adverse events.

### Quality assessment

The methodological quality of eligible studies was rated via the MINORS scale. 15 studies (55.6%) were of moderate quality (MINORS score of 9 - 12), and 12 (44.4%) were of high quality (MINORS score of 13 - 16). The specific quality scores are provided in **Supplementary Table S3**.

### Tumor response

**Figure 2:**
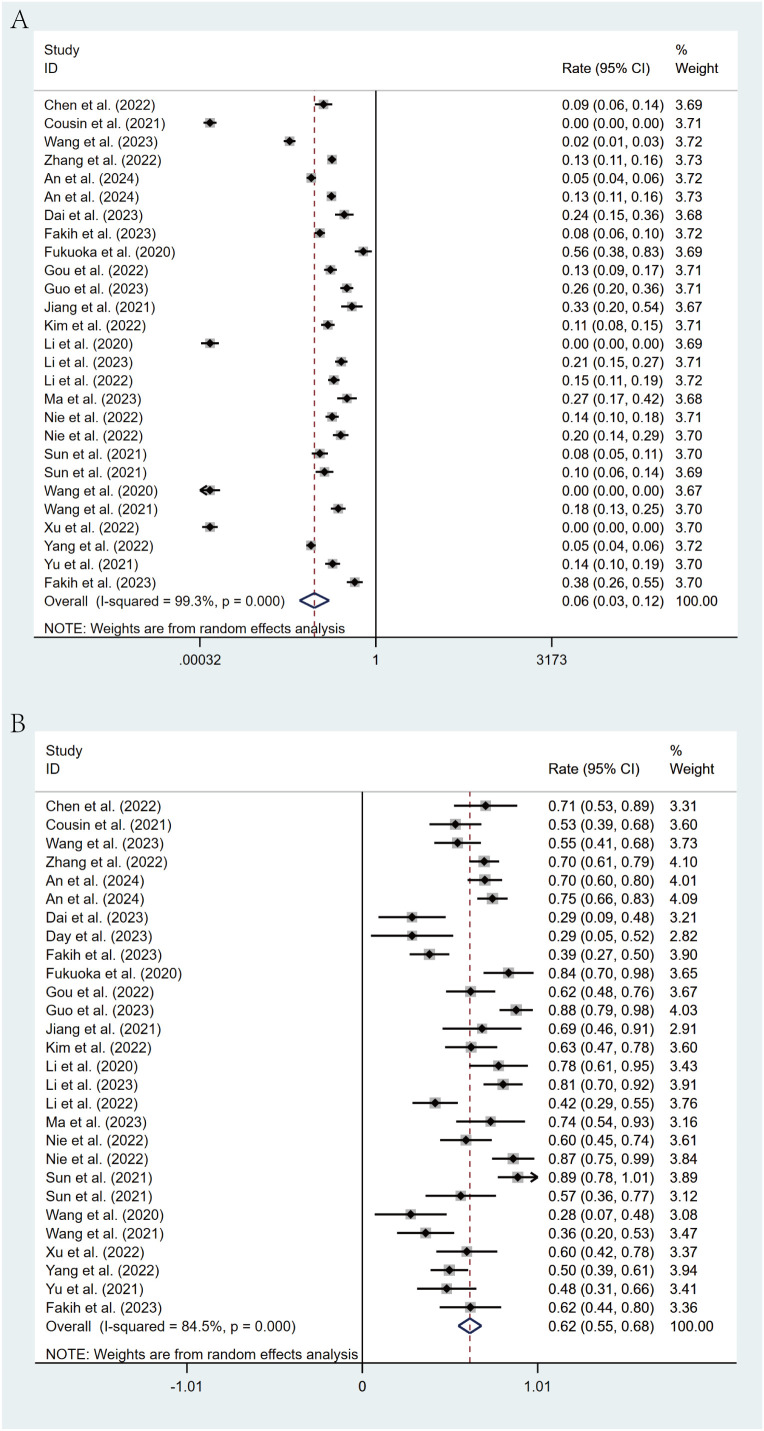
Forest plot for pooled results of ORR **(A)** and DCR **(B)** in mCRC patients treated with regorafenib/fruquintinib plus PD-1/PD-L1.

All studies reported the efficacy of regorafenib or fruquintinib combined with PD-1/PD-L1 for the treatment of mCRC. 24 studies reported ORR. With marked heterogeneity (I² = 99.3%, p < 0.001), the pooled analysis of ORR was implemented utilizing a random-effects model. The combined ORR was 6% (95% CI: 3% - 12%, p < 0.001) (**Figure 2A**). Moreover, 25 studies reported DCR, which also exhibited notable heterogeneity (I² = 84.5%, p < 0.001). A random-effects model was leveraged, and the combined DCR was 62% (95% CI: 55% - 68%, p < 0.001) (**Figure 2B**).

### Survival analysis

**Figure 3:**
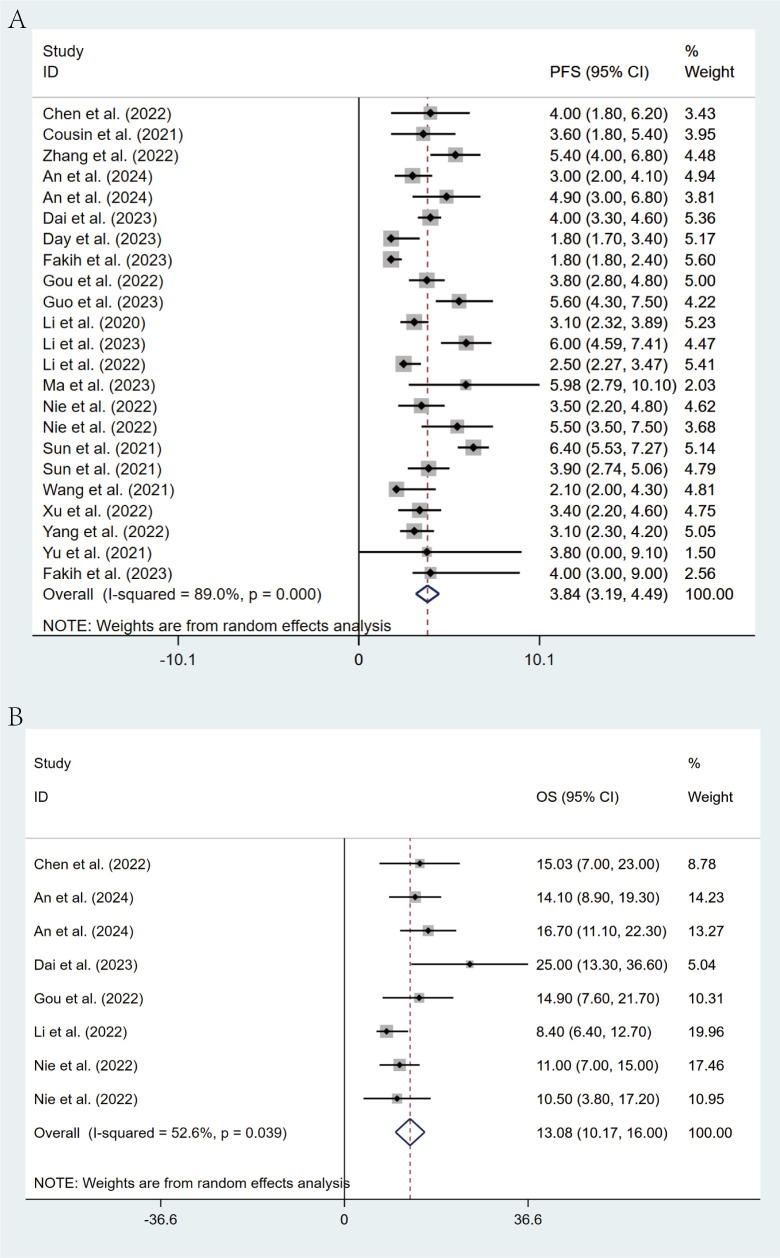
Forest plot for pooled results of PFS **(A)** and OS **(B)** in mCRC patients receiving regorafenib/fruquintinib plus PD-1/PD-L1.

Of the included studies, 20 studies reported PFS in patients, while 6 studies reported OS. Through a random-effects model (I² = 89.0%, p < 0.001), the pooled median PFS was 3.84 months (95% CI: 3.19 - 4.49, p < 0.001), as shown in **Figure 3A**. For OS, the same random-effects model was applied (I² = 52.6%, p = 0.039), and the pooled median OS was 13.08 months (95% CI: 10.17 - 16.00, p < 0.001), as illustrated in **Figure 3B**.

### Safety analysis

The most common grade 3–4 AEs linked to the combination of regorafenib/fruquintinib and PD-1/PD-L1 inhibitors in mCRC were analyzed, and the incidence rate was 21% (95% CI: 15%-28%) (**Table 2**). The most frequent AEs were hypertension, hand-foot skin reaction (HFSR), elevated ALT/AST and liver dysfunction, and hand-foot syndrome, with incidence rates of 24% (95% CI: 18%-30%), 20% (95% CI: 9%-41%), 20% (95% CI: 15%-26%), and 18% (95% CI: 12%-26%), respectively. The most common grade 3–4 hematologic toxicities encompassed anemia (8%, 95% CI: 4%-16%), leukopenia (8%, 95% CI: 3%-20%), neutropenia (6%, 95% CI: 3%-13%), and thrombocytopenia (5%, 95% CI: 2%-9%). Other frequently observed grade 3–4 AEs included fatigue, oral mucositis, elevated bilirubin, increased lipase levels, rash, reactive skin capillary endothelial proliferation, and fever. Most AEs were grade 1–2 and treatable. Subgroup analysis showed similar rates of grade 3–4 AEs, at 23% (95% CI: 16%-31%) and 19% (95% CI: 9%-36%) in the fuquintinib and regorafenib groups, respectively, and statistically significant differences were not noted across groups (**Supplementary Figure S1**). The most frequently reported AEs leading to treatment discontinuation or dose modification were dermatologic toxicities, such as hand-foot skin reaction, palmar-plantar erythrodysesthesia, rash, and maculopapular rash, followed by hepatic dysfunction and hypertension. irAEs occurred less frequently than with regorafenib or fruquintinib monotherapy. However, several studies still reported severe irAEs, primarily including hepatic dysfunction, dermatologic toxicities, colitis, and immune-mediated pneumonitis. One treatment-related death due to immune myocarditis was documented (**Supplementary Table S2**).

**Table 2:** Meta-analysis of adverse events.

AE	Study (n)	(I^2^,p)	Rate, 95%CI
Grade 3-4	20	82.4%, p<0.01	0.21 (0.15, 0.28)
Hypertension	15	0%, p=0.76	0.24 (0.18, 0.30)
Proteinuria	11	0%, p=1.0	0.01 (0.00, 0.05)
Palmar-plantar erythrodysesthesia syndrome	6	69%, p<0.01	0.20 (0.09, 0.41)
Diarrhea	12	14%, p=0.13	0.02 (0.00, 0.06)
Fatigue	13	0%, p=1.0	0.05 (0.02, 0.09)
Oral mucositis	8	0%, p=1.0	0.02 (0.01, 0.09)
AST/ALT increased/Liver dysfunction	16	31%, p=0.11	0.20 (0.15, 0.26)
Blood bilirubin increase	7	0%, p=1.0	0.07 (0.04, 0.14)
Lipase increase	5	0%, p=0.87	0.07 (0.03, 0.16)
Rash	10	0%, p=0.93	0.13 (0.08, 0.21)
Fever	11	0%, p=1.0	0.02 (0.00, 0.06)
Platelet count decreased	10	0%, p=1.0	0.05 (0.02, 0.09)
Neutropenia	7	0%, p=1.0	0.06 (0.03, 0.13)
Leukopenia	5	0%, p=0.97	0.08 (0.03, 0.20)
Anorexia	10	0%, p=1.0	0.01 (0.00, 0.04)
Anemia	6	0%, p=0.90	0.08 (0.04, 0.16)
Reactive cutaneous capillary endothelial proliferation	4	0%, p=1.0	0.11 (0.03, 0.35)
Hypothyroidism	13	14%, p=0.30	0.00 (0.00, 0.01)

### Subgroup analysis

**Figure 4:**
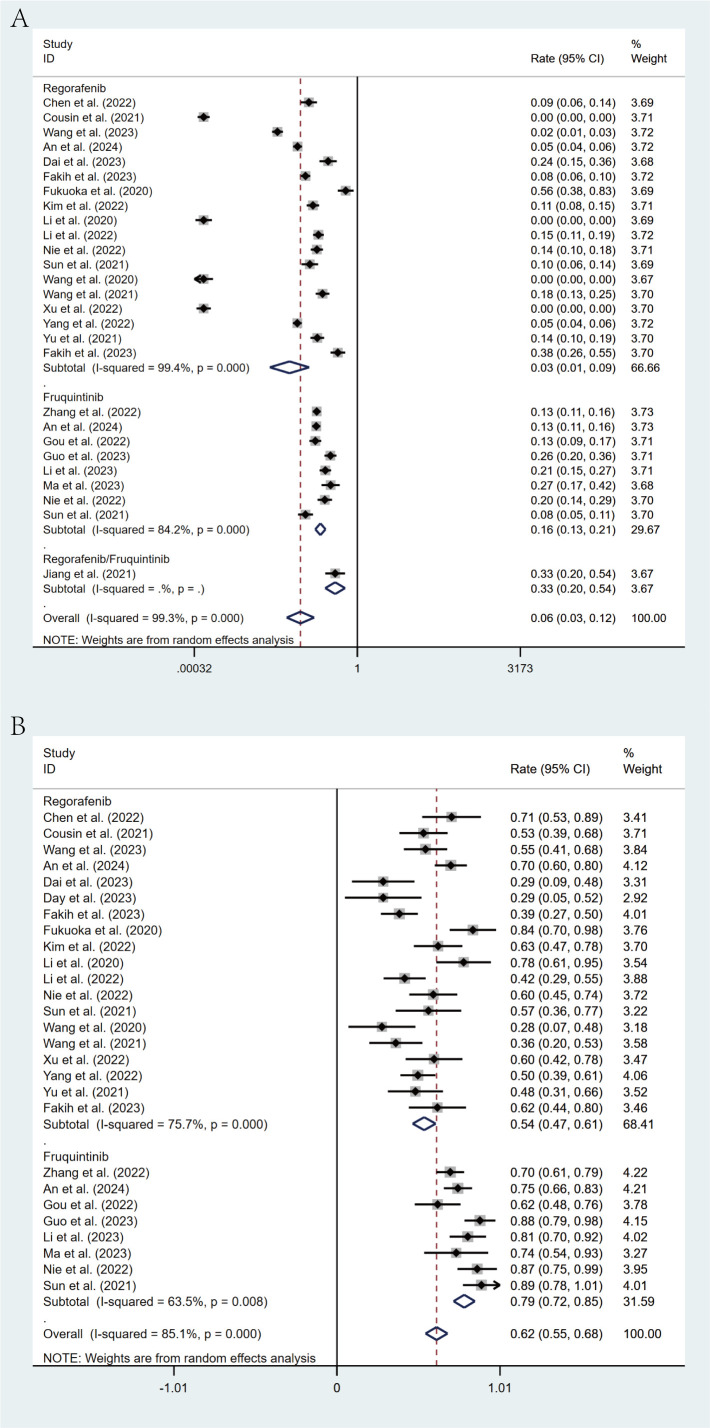
Forest plot of the subgroup analysis for ORR **(A)** and DCR **(B)** across different drug combination treatment groups (Regorafenib vs Fruquintinib).

**Figure 5:**
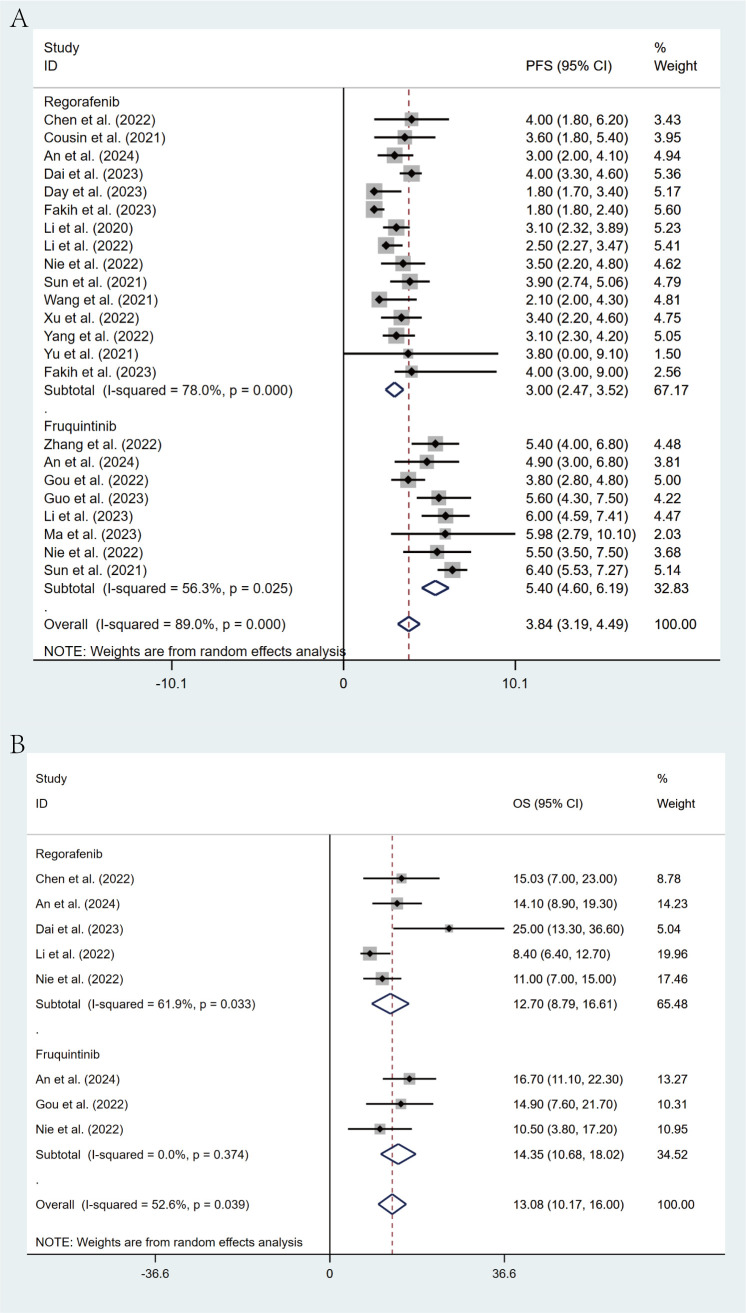
Forest plot of the subgroup analysis for PFS **(A)** and OS **(B)** across different drug combination treatment groups (Regorafenib vs Fruquintinib).

Comprehensive subgroup analyses were carried out to evaluate the influence of various clinical factors on treatment efficacy. The meta-analysis revealed significant differences in efficacy between treatment regimens. The fruquintinib-based regimen demonstrated superior outcomes, with an ORR of 16% (95% CI: 13%-21%) and a DCR of 79% (95% CI: 72%-85%), in comparison to the regorafenib-based regimen, which yielded an ORR of 3% (95% CI: 1%-9%) and a DCR of 54% (95% CI: 47%-61%). The lack of overlap in CIs indicates statistical significance (**Figures 4A, B**). This observed efficacy advantage was further substantiated by survival outcomes: the fruquintinib-based group achieved a significantly longer median PFS of 5.40 months (95% CI: 4.60-6.19) in contrast to 3.00 months (95% CI: 2.47-3.52) in the regorafenib group. Although median OS was numerically longer in the fruquintinib group (14.35 months [95% CI: 10.68-18.02] vs. 12.70 months [95% CI: 8.79-16.61]), the partial overlap in CIs suggests that the difference was not statistically significant (**Figures 5A, B**).

**Figure 6:**
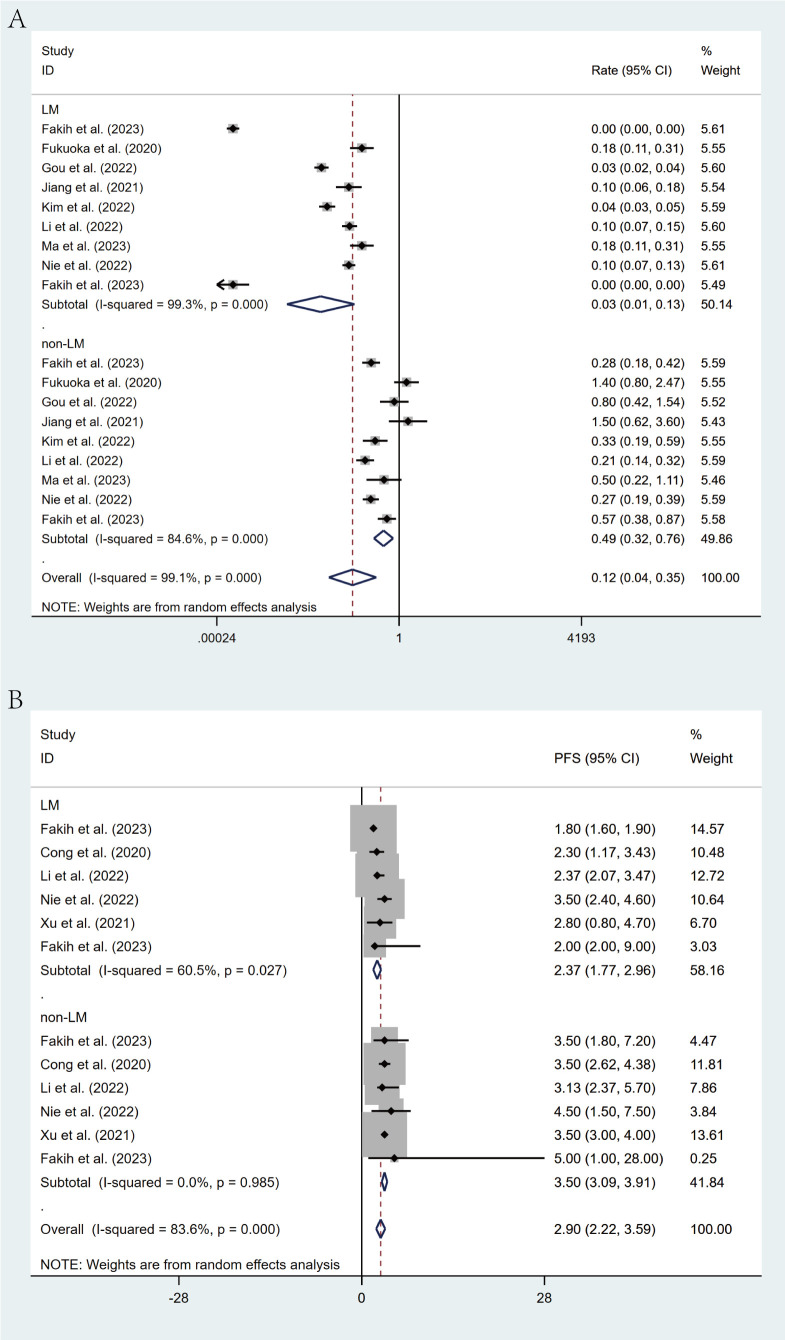
Forest plot of the subgroup analysis for ORR **(A)** and PFS **(B)** across different groups (LM vs non-LM).

Stratification based on liver metastasis status revealed a marked influence on treatment efficacy. Patients with liver metastases demonstrated significantly poorer outcomes, with an ORR of 3% (95% CI: 1%-13%) in contrast to 49% (95% CI: 32%-76%) in patients without liver involvement. Similarly, median PFS was considerably shorter among those with liver metastases (2.37 months [95% CI: 1.77-2.96]) than in those without (3.50 months [95% CI: 3.09-3.91]). The non-overlapping CIs for both ORR and PFS confirm the statistical significance of these findings (**Figures 6A, B**).

**Figure 7:**
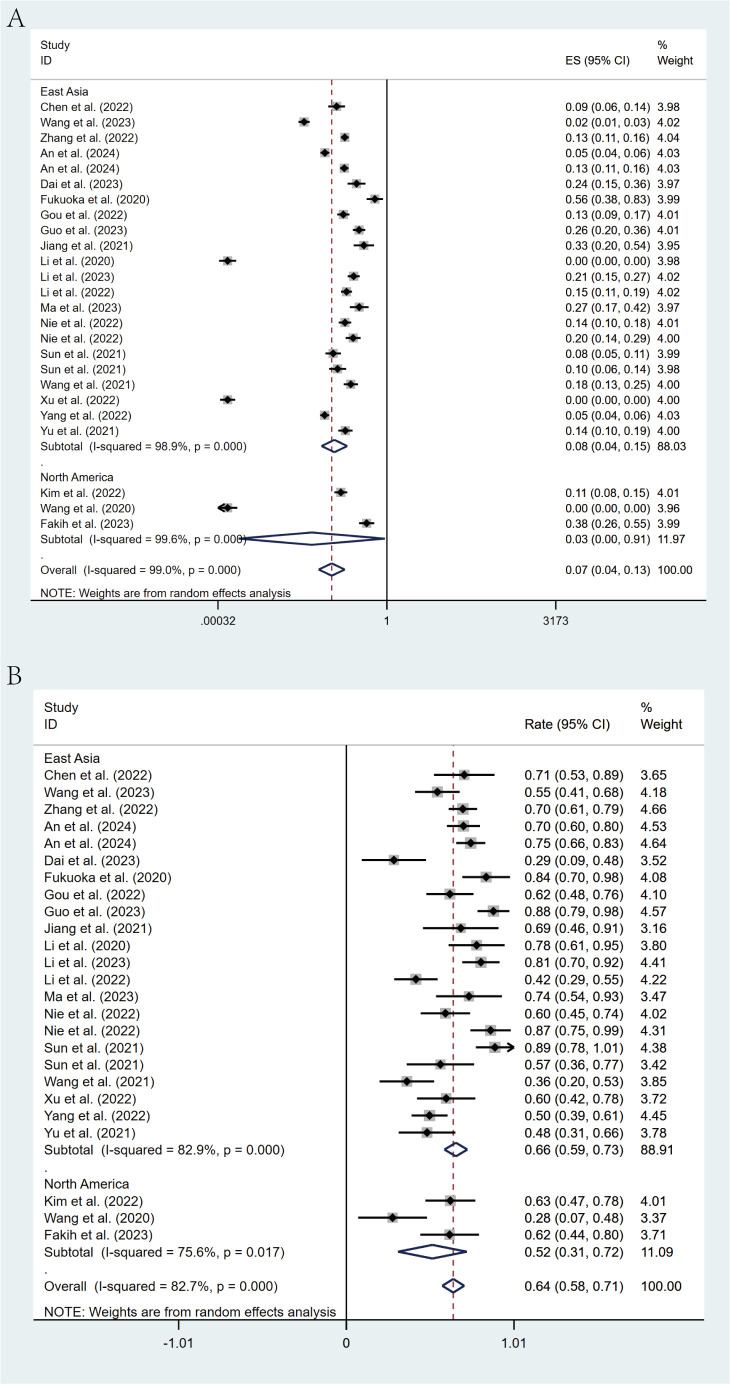
Forest plot of the subgroup analysis for ORR **(A)** and DCR **(B)** across different countries (East Asia vs North America).

Geographic subgroup analysis revealed that patients from East Asia demonstrated an ORR of 8% (95% CI: 4%-15%) and a DCR of 66% (95% CI: 59%-73%), whereas North American cohorts exhibited an ORR of 3% (95% CI: 0%-91%) and a DCR of 52% (95% CI: 31%-72%) (**Figures 7A, B**).

Subgroup analysis by type of ICIs indicated that studies employing PD-1 inhibitors (n = 25) reported a median PFS of 3.85 months (95% CI: 3.09-3.91), an ORR of 7% (95% CI: 4%-13%), and a DCR of 62% (95% CI: 55%-69%) (**Supplementary Figures S2**, **S3**). In contrast, the single study utilizing a PD-L1 inhibitor reported a median PFS of 3.6 months, an ORR of 0%, and a DCR of 23%. According to the subgroup analysis by previous treatment lines (< 50% vs >50% of patients receiving ≥ third-line treatment), the ORR (15% vs 14%), DCR (66% vs 63%), PFS (3.90 vs 3.92 months) and OS (13.01 vs 13.57 months) of the <50% group and the >50% group were consistent (**Supplementary Figures S4**, **S5**). According to the subgroup analysis by study design types, there was little difference in ORR (5% vs 10%), DCR (63% vs 62%) and PFS (4.08 months vs 3.06 months) between retrospective studies and prospective studies (**Supplementary Figures S6**, **S7**).

### Sensitivity analysis

**Figure 8:**
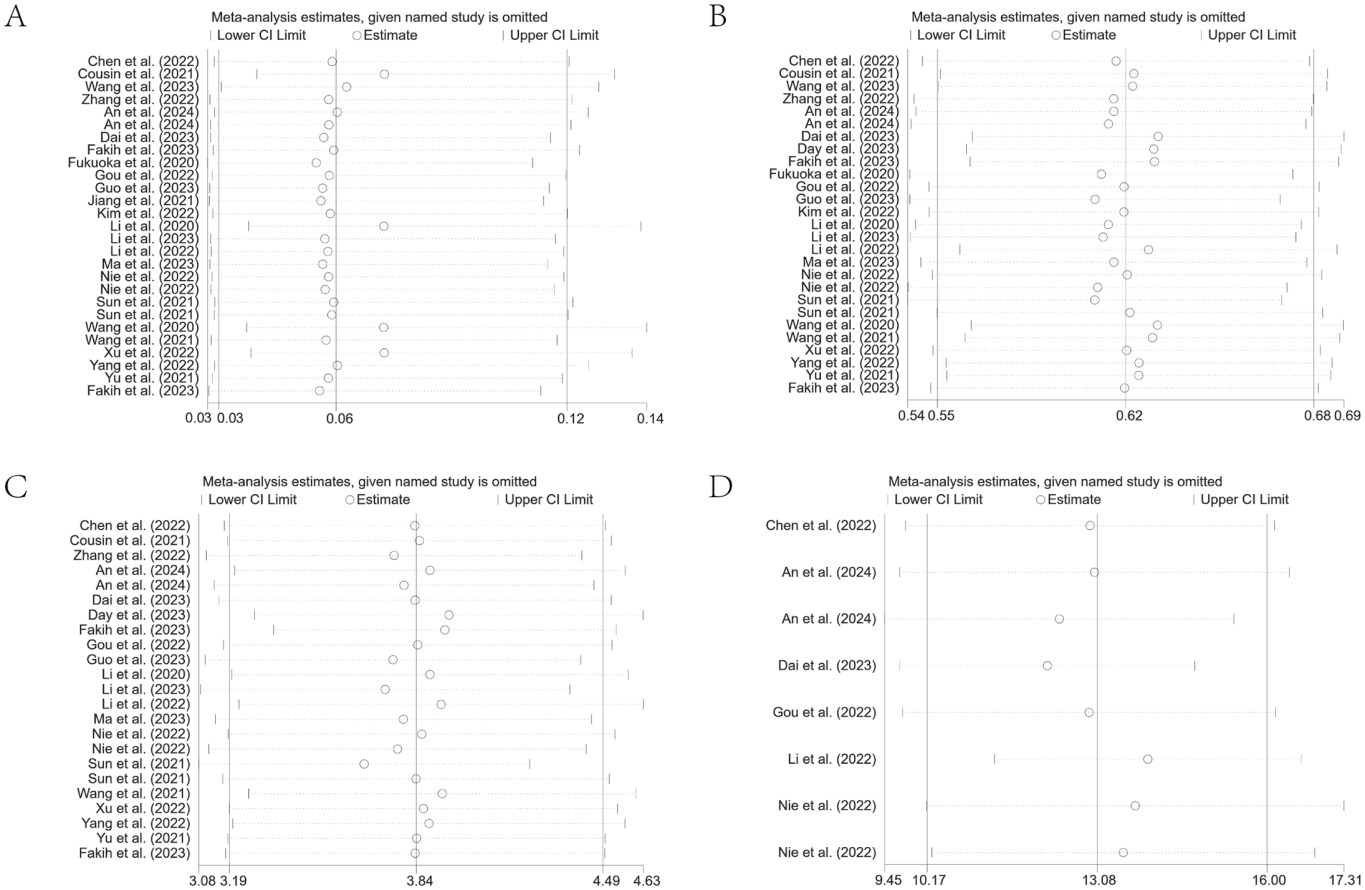
Leave-one-out sensitivity analysis of ORR **(A)**, DCR **(B)**, PFS **(C)**, and OS **(D)** for patients receiving regorafenib/fruquintinib plus PD-1/PD-L1.

The robustness of the summary results was determined through a sensitivity analysis by systematically eliminating each study. The findings indicated that none of the summary results, nor their 95% CIs, were significantly affected by the exclusion of any single study. This suggested generally robust and reliable results of our meta-analysis. The results are detailed in **Figures 8A–D**.

### Publication bias

**Figure 9:**
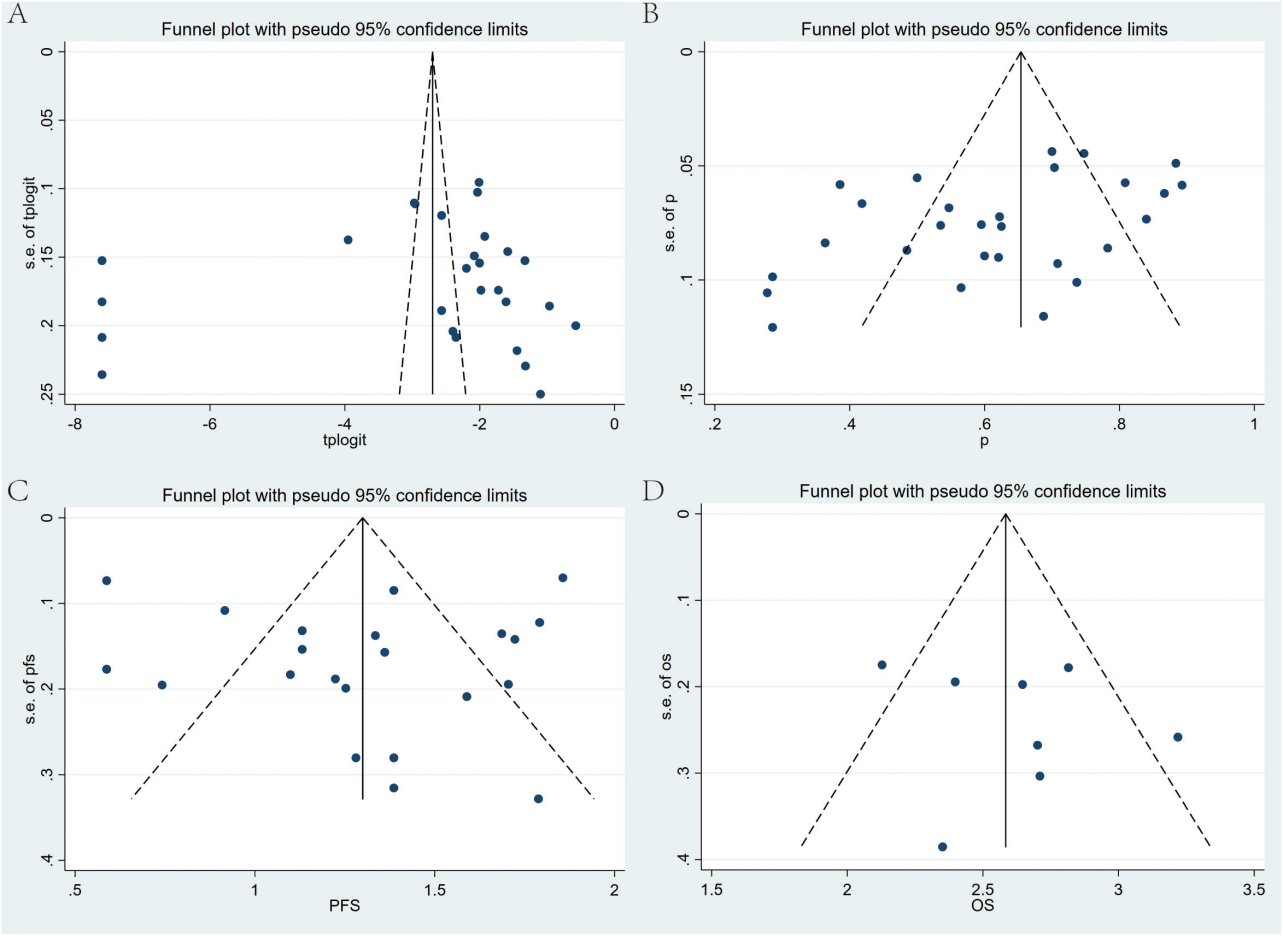
Begg’s funnel plots for publication bias test with pseudo 95% confidence limits. **(A)** ORR, **(B)** DCR, **(C)** PFS, and **(D)** OS of Regorafenib/Fruquintinib in combination with PD-1/PD-L1 for mCRC.

Publication bias was detected through Egger’s test and Begg’s test (**Figures 9A–D**). The results indicated significant publication bias only for DCR (Egger’s test: p = 0.02), while discernible bias was not found for other metrics. For the observed publication bias in DCR, the trim-and-fill method was further applied for adjustment. Before trimming, the DCR was 62% (95% CI: 55%-68%, p < 0.001); and after adjustment, the p-value remained less than 0.001, with no reversal in results, indicating relative stability and reliability of the findings (**Supplementary Figure S8**).

## Discussion

Our study is the first systematic evaluation of the efficacy and safety of regorafenib/fruquintinib plus PD-1/PD-L1 in mCRC through a meta-analysis. By incorporating data from 26 studies involving 1409 patients, our aggregated analysis reveals that the combination therapy demonstrates positive efficacy across ORR, DCR, PFS, and OS, with manageable safety. Specifically, the ORR for the combination treatment was 6%, and the DCR was as high as 62%, indicating considerable clinical benefit for a substantial proportion of patients. As for survival benefits, OS reached 13.08 months, while the PFS was 3.84 months, highlighting the positive role of combination therapy in prolonging patient survival. The incidence of grade 3–4 AEs was 21%, which is acceptable, suggesting favorable safety. This study provides important evidence-based support for treatment choices in mCRC sufferers.

At present, the standard third-line treatment options for mCRC mainly comprise three regimens: regorafenib, fruquintinib, and trifluridine as monotherapy. Historical clinical data indicate that regorafenib monotherapy, compared to placebo, yields an ORR of 1% and a median OS of 6.4 months in mCRC individuals (4). The fruquintinib monotherapy, compared to placebo, exhibits an ORR of 4.7% and a median OS of 9.3 months in individuals with mCRC (5), whereas trifluridine monotherapy yields an ORR of only 1.6% and a median OS of 7.1 months (6). In the meta-analysis conducted by Walter et al. (7) on third-line treatment for mCRC, the OS for monotherapy ranged from 6.4 to 7.9 months. This combination strategy yields superior outcomes in terms of ORR and median OS when compared to historical monotherapy data, indicating that the integration of anti-angiogenic agents with ICIs may offer a more effective treatment approach, thereby providing novel therapeutic options for the mCRC population.

In our meta-analysis, subgroup analyses revealed significant differences in treatment efficacy across various treatments, patient populations, and disease characteristics, providing insights for personalized therapeutic approaches. First and foremost, subgroup analysis demonstrated a notably higher ORR of 16% versus 3% and DCR of 79% versus 54% with fruquintinib in contrast to regorafenib, indicating enhanced antitumor activity, which may be attributed to differences in their pharmacologic profiles. Fruquintinib’s selective inhibition of VEGFR1–3 may lead to better angiogenesis control and improved tolerability in contrast to regorafenib’s broader kinase inhibition (18). The prolonged PFS in the fruquintinib group (5.40 vs. 3.00 months) further supports its clinical advantage in delaying disease progression. However, the absence of a statistically significant OS benefit (14.35 vs. 12.70 months) may be influenced by confounding factors like subsequent therapies or the relatively short follow-up duration (with only 6 of 26 studies providing OS data), highlighting the need for long-term survival data. In patients with liver metastases, a markedly reduced ORR (3% vs. 49%) and shorter PFS (2.37 vs. 3.50 months) were observed, which aligns with research indicating that liver metastasis possibly suppresses antitumor immune responses (19). Therefore, liver metastasis could serve as a predictive marker for treatment efficacy, guiding the development of individualized treatment strategies for this subgroup, including the potential for increased use of local liver therapies. Yu et al. (19) also demonstrated that radiotherapy targeting liver metastases can reshape the liver immune microenvironment, thereby enhancing the effects of antitumor immunotherapy, which provides valuable insights for clinical decision-making. Furthermore, the subgroup analysis also revealed significant efficacy advantages in East Asian patients (ORR = 15% vs. 3% in North America), potentially reflecting multifactorial influences, including genetic polymorphisms in drug metabolism, regional differences in supportive care, or variability in baseline tumor characteristics (4, 20). However, the extremely broad CI for the North American ORR (0%-91%) precludes definitive conclusions, reflecting the urgent need for geographically balanced trials. Our study differs from previous meta-analyses regarding the efficacy of ICIs in mCRC. Firstly, in terms of the scope, our study specifically focuses on anti-angiogenic agents (regorafenib/fruquintinib) combined with ICs, whereas the study by Zhang et al. (21) encompassed all treatment regimens involving PD-1/PD-L1, leading to a broader and less focused research focus. Secondly, regarding study quality, although the meta-analysis by Zeng et al. (22) incorporated only randomized controlled trials (RCTs), it ultimately incorporated just three studies, these studies compared PD-1/PD-L1 monotherapy with various control groups (including regorafenib monotherapy, best supportive care, and chemotherapy), the inconsistency in control group treatments may have introduced heterogeneity in the results. In contrast, our study included a greater number of studies, including several real-world data sources from China, thereby enhancing the representativeness of the findings. Thirdly, regarding the assessment criteria, the meta-analysis by Li et al. (23) focused on short-term therapeutic response indicators such as ORR and DCR, whereas our study evaluated not only tumor response rates but also included PFS and OS to conduct a comprehensive survival analysis. Notably, in the subgroup analysis, we considered factors such as liver metastasis status, different combination therapies, and different national populations, thereby providing more comprehensive evidence for clinical practice. In summary, this study offers a more targeted clinical reference for combination therapy strategies in mCRC through a more focused research scope, a larger sample size, more comprehensive assessment criteria, and more detailed subgroup analyses. These findings hold significant implications for optimizing treatment strategies and predicting patient prognosis.

In terms of predictive biomarkers, tumor mutational burden (TMB) serves as a biological marker for immunotherapy across various solid tumors, with TMB-high (TMB-H) identified as a biomarker for pembrolizumab treatment in solid tumors. TMB-H potentially reflects the tumor’s capacity to generate neoantigens (24, 25). However, the availability of TMB data was limited in our meta-analysis, with only 19.2% of studies providing relevant information, and significant inconsistencies were observed in assessment methods and threshold definitions. Among the limited data available, only Fukuoka et al. (14) reported that high TMB was linked to prolonged median PFS, though small sample sizes and methodological heterogeneity necessitate caution in interpreting these results. Research suggests that PD-L1-positive populations exhibit higher levels of immunosuppressive markers like FOXP3 and CSF1R, indicating that ICs combined with regorafenib (which targets immunosuppressive cells) may offer potential benefits for PD-L1-positive patients (26). However, Fukuoka et al. (14) found no clear link between PD-L1 and treatment efficacy with this combination in the mCRC population. Other predictive biomarkers demonstrated varying degrees of correlation with clinical outcomes. Immune-related biomarkers, including CD8 expression and regulatory T-cell levels, were associated with improved clinical outcomes in several studies (27, 28). Concerning metastatic patterns, both liver and lung metastasis status were confirmed as significant predictive factors (28). Additionally, specific gene mutations, such as those in ERBB2/3 (29), as well as various laboratory parameters (30), exhibited potential predictive value, as outlined in the **Supplementary Materials** (**Supplementary Table S4**). Nevertheless, the heterogeneity in assessment methods and occasionally contradictory results complicate the ability to draw definitive conclusions.

The synergistic mechanism of anti-angiogenic TKIs combined with ICIs involves multiple dimensions of tumor biology. Primarily, anti-angiogenic TKIs (regorafenib/fruquintinib) facilitate the normalization of tumor vascular by inhibiting VEGF/VEGFR pathway, thereby attenuating hypoxic conditions within the tumor microenvironment, enhancing T cell infiltration, and ultimately potentiating immunotherapeutic efficacy (31, 32). Additionally, VEGF inhibition significantly diminishes the proliferation of immunosuppressive regulatory T cells (Tregs) and restricts the expansion of myeloid-derived suppressor cells (MDSCs), while concurrently promoting dendritic cell (DC) maturation and function (33–35). Furthermore, these anti-angiogenic agents effectively reduce tumor-associated macrophages (TAMs) by inhibiting colony-stimulating factor 1 receptor, and specifically suppressing the p38 kinase/CREB1/KLF4 signaling axis, and facilitating the critical conversion of immunosuppressive M2-like TAMs toward immunostimulatory M1-like phenotypes (12). Furthermore, TKIs upregulate the expression of intratumoral chemokines (particularly CXCL10), which substantially increases the recruitment and infiltration of CD8+ T cells (36). Collectively, these complementary mechanisms orchestrate the transformation of the tumor immune microenvironment from an immunosuppressive to an immunosupportive state (32), consequently enhancing the efficacy of anti-PD-1/PD-L1 immunotherapy and generating durable anti-tumor immune responses (13, 37).

The findings of this study hold significant clinical implications. Firstly, this research confirms that anti-angiogenic agents combined with PD-1 demonstrate certain therapeutic efficacy in mCRC individuals, thereby providing a new treatment option for this patient population. Additionally, MSS CRC accounts for 95% of advanced-stage patients, and single-agent immunotherapy has proven to be largely ineffective in this group (38). This study offers novel therapeutic strategies for patients who are unresponsive to conventional immunotherapy. Furthermore, it identifies differences in the treatment benefits among various subgroups of patients. Patients without liver metastases, particularly those receiving fruquintinib-based combination therapy, obtained more benefits. These findings offer a basis for clinicians to develop personalized treatments. Specifically, the combination therapy is most beneficial for patients without liver metastases. This aligns with the results of several studies, such as the REGONIVO trial, which also demonstrated that in comparison to individuals with liver metastases (15.4%), those without liver metastases had a considerably higher ORR (58.3%) (14). Moreover, this study demonstrates that the safety of the combined therapy is manageable. The incidence of grade 3–4 AEs was 21%, primarily including hypertension, hand-foot syndrome with sensory abnormalities, and liver function abnormalities, which are consistent with the safety profiles reported in previous studies, with no new safety concerns observed. The foregoing findings offer a valuable reference for the safe application of this combination therapy in clinical practice.

However, our study has limitations. First, most eligible studies were retrospective or single-arm, and there were no large-scale prospective RCTs. This may affect the level of evidence and could lead to an overestimation of treatment efficacy. Second, the samples were relatively limited, with 20–50 cases enrolled in most studies, which possibly impacted the statistical power and stability of our results. Third, there was heterogeneity in the study populations. Baseline characteristics of patients, such as ECOG score, prior lines of treatment, and metastatic sites, varied across studies. Fourth, significant variations in the immunotherapy agents used across the included studies represent a notable limitation of our meta-analysis. Twenty-five studies employed various PD-1 inhibitors, including nivolumab, pembrolizumab, camrelizumab, sintilimab, toripalimab, and tislelizumab, while one study utilized a PD-L1 inhibitor, avelumab. The heterogeneous distribution of these agents hindered the ability to perform meaningful comparative analyses of specific immunotherapeutic drugs. Fifth, the starting doses of anti-angiogenic agents were not consistent across studies. Sixth, the follow-up periods varied among studies, which may affect the comparability of survival data. Seventh, as this meta-analysis only included English-language publications, there is a potential risk of language bias, which may affect the representativeness of the study results.

In view of the aforementioned limitations, future research should be conducted from multiple perspectives, including clinical practice, treatment optimization, biomarker screening, and mechanisms. First, concerning clinical research, the majority of current studies are retrospective or single-arm; future large-scale, prospective RCTs are necessary to validate the efficacy and safety of combination therapies, such as the ongoing Phase III study of pembrolizumab plus lenvatinib (NCT04776148). In particular, specialized clinical trials are needed to assess the differences in efficacy between specific subgroups, such as patients using different treatment regimens and with liver metastases. Second, in optimizing treatment strategies, it is essential to determine the optimal dosing and sequencing of combination therapies, explore selection criteria for different PD-1/PD-L1, and for patients with liver metastases, and study the best combination of local treatments (such as ablation) with systemic therapies. Third, there is a need for in-depth exploration of predictive biomarkers of efficacy, including immune-related markers (such as PD-L1 expression and CD8+ T cell infiltration, regulatory T-cell levels), specific gene mutations, angiogenesis-related markers (such as IL-8 and VEGF), their relationship with treatment response, as well as the relationship between the microenvironment of different metastatic sites and treatment outcomes. Finally, mechanistic studies are essential, including elucidating the synergistic mechanisms between anti-angiogenic agents and ICIs and the molecular mechanisms behind efficacy differences in patients with metastases to various parts, particularly those with liver metastasis. These research directions will help further optimize treatment strategies and identify patients who may benefit most, ultimately improving treatment outcomes for mCRC patients. In conclusion, our study offers important fresh insights into the management of mCRC. Further multi-faceted investigations are warranted to enhance treatment efficacy and optimize therapeutic strategies.

## Conclusion

In conclusion, this meta-analysis proves the efficacy and safety of regorafenib/fruquintinib combined with PD-1/PD-L1 in the management of mCRC. This combination therapy significantly improves ORR and DCR, while also extending survival. The observed incidence of grade 3–4 AEs indicates that the safety of this combination therapy is controllable. This combination therapy offers a new therapeutic option for mCRC patients. However, available clinical data were scarce. Consequently, large, multi-center RCTs are needed to verify our findings, optimize predictive biomarkers of efficacy, and refine treatment strategies to better guide clinical practice.

## Data Availability

The original contributions presented in the study are included in the article/**Supplementary Material**. Further inquiries can be directed to the corresponding authors.

## Supplementary material

Supplementary Figure 1 Forest plot of subgroup analysis comparing the rates of grade 3–4 adverse events between regorafenib and fruquintinib treatment groups.

Supplementary Figure 2 Forest plot for pooled results of DCR **(A)** and ORR **(B)** in mCRC patients treated with regorafenib/fruquintinib plus PD-1.

Supplementary Figure 3 Forest plot for pooled results of PFS in mCRC patients treated with regorafenib/fruquintinib plus PD-1.

Supplementary Figure 4 Forest plot of the subgroup analysis for ORR **(A)** and DCR **(B)** across different lines of treatment (≥ third-line <50% vs. ≥ third-line >50%).

Supplementary Figure 5 Forest plot of the subgroup analysis for PFS **(A)** and OS **(B)** across different lines of treatment (≥ third-line <50% vs. ≥ third-line >50%).

Supplementary Figure 6 Forest plot of the subgroup analysis for ORR **(A)** and DCR **(B)** across different study designs (Retrospective study vs. Prospective study).

Supplementary Figure 7 Forest plot of the subgroup analysis for PFS **(A)** and OS **(B)** across different study designs (Retrospective study vs. Prospective study).

Supplementary Figure 8 Publication bias assessment for disease control rate (DCR) using Egger’s test **(A)** and trim-and-fill method **(B)**.
