# Supplementary figures and images for: The efficacy and safety of regorafenib/fruquintinib combined with PD-1/PD-L1 for metastatic colorectal cancer: a meta-analysis based on single-arm studies
Source: Front Immunol. 2025 May 29;16:1579293. doi: 10.3389/fimmu.2025.1579293 (PMC12159013; doi:10.3389/fimmu.2025.1579293)

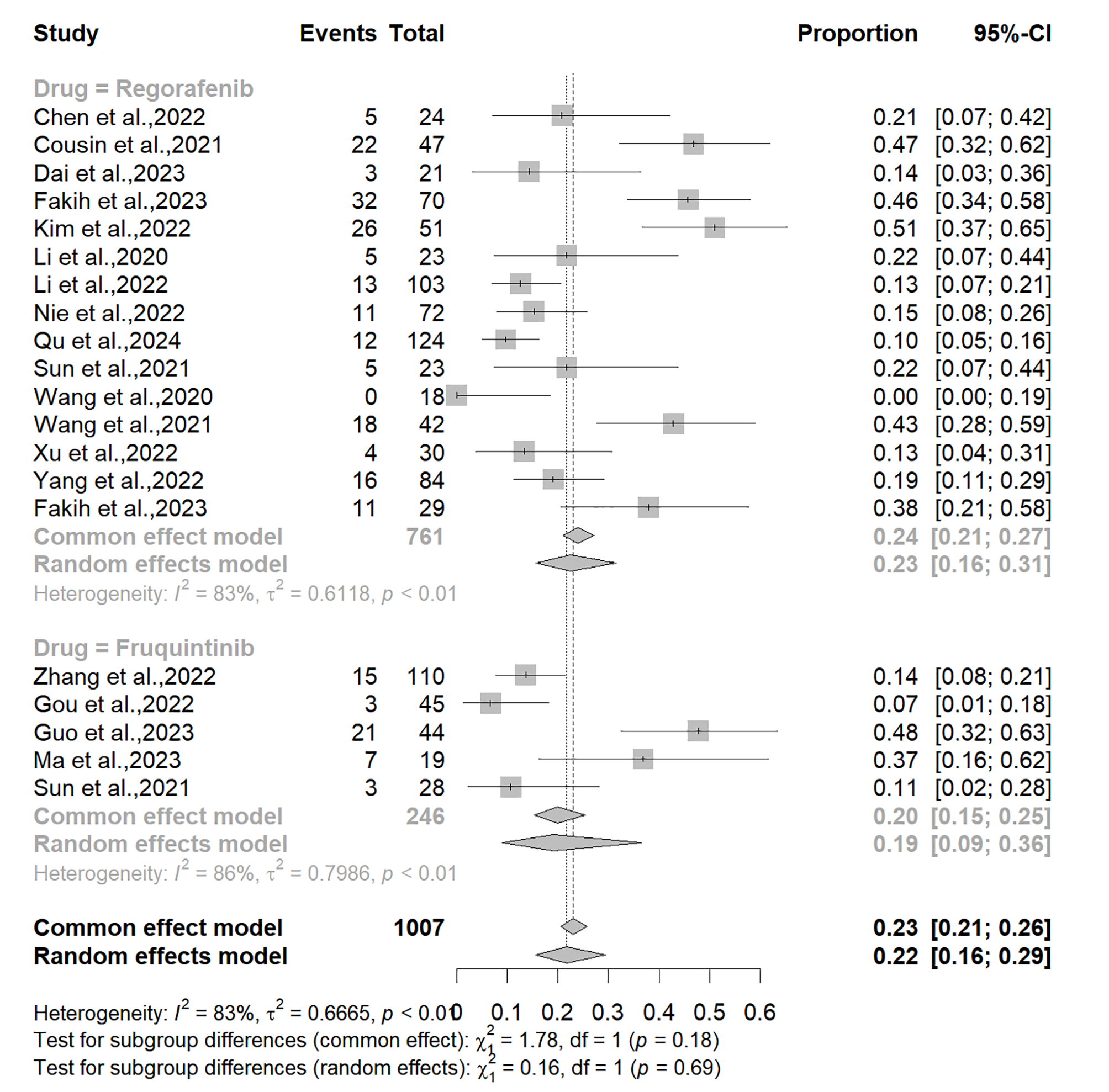

Supplement: Supplementary Figure 1 — Forest plot of subgroup analysis comparing the rates of grade 3–4 adverse events between regorafenib and fruquintinib treatment groups. [file Image1.tiff]

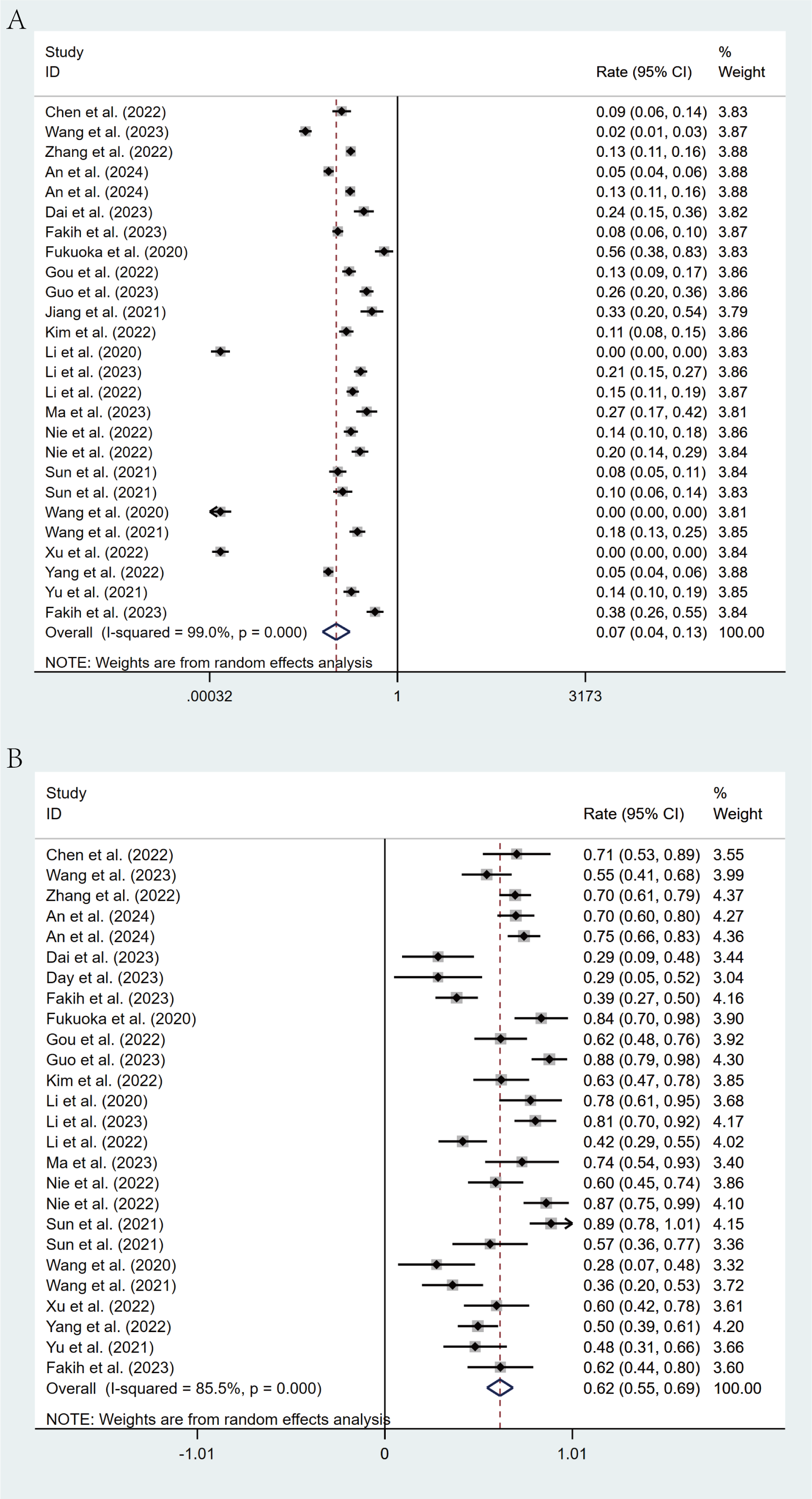

Supplement: Supplementary Figure 2 — Forest plot for pooled results of DCR (A) and ORR (B) in mCRC patients treated with regorafenib/fruquintinib plus PD-1. [file Image2.tif]

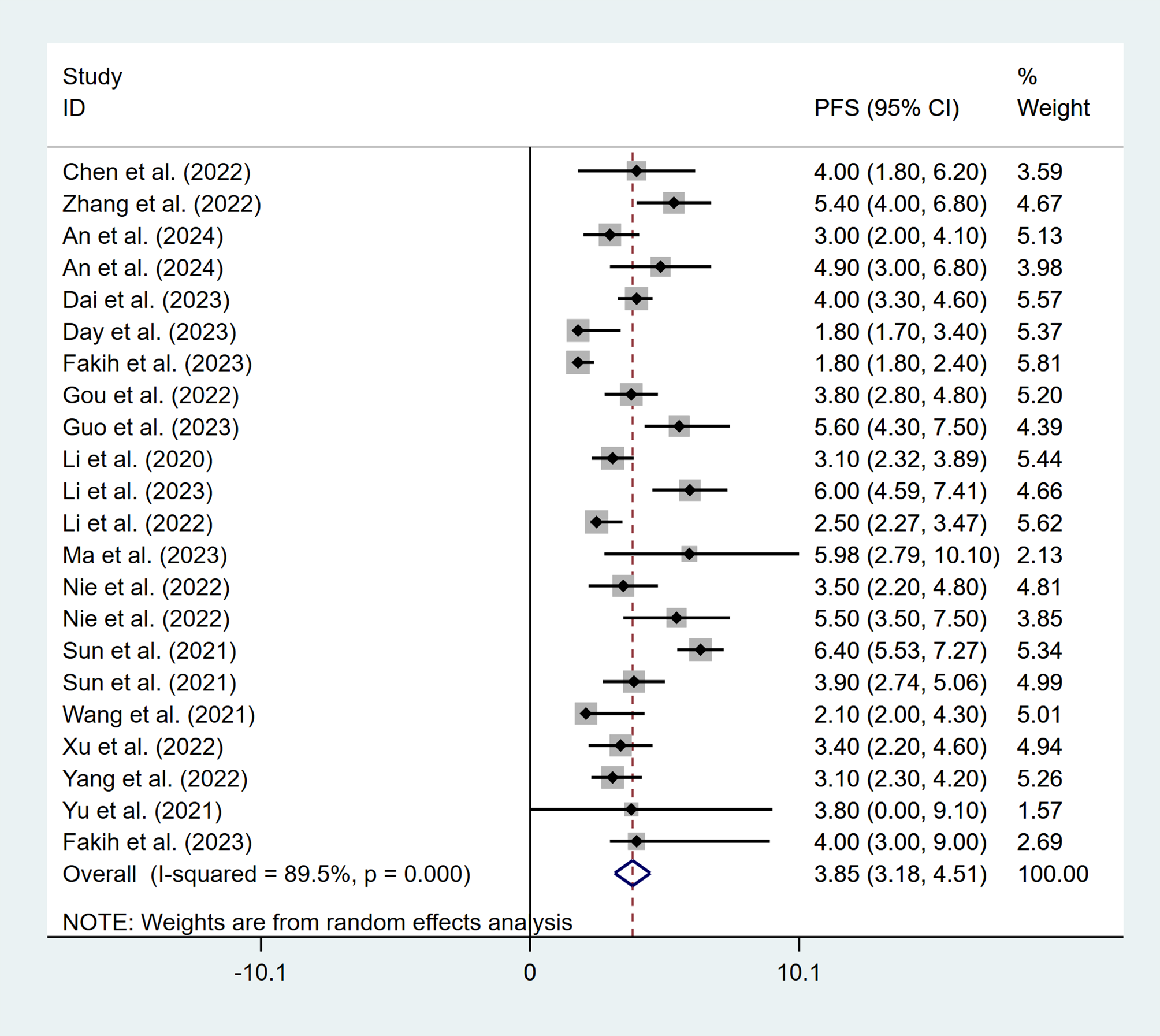

Supplement: Supplementary Figure 3 — Forest plot for pooled results of PFS in mCRC patients treated with regorafenib/fruquintinib plus PD-1. [file Image3.tif]

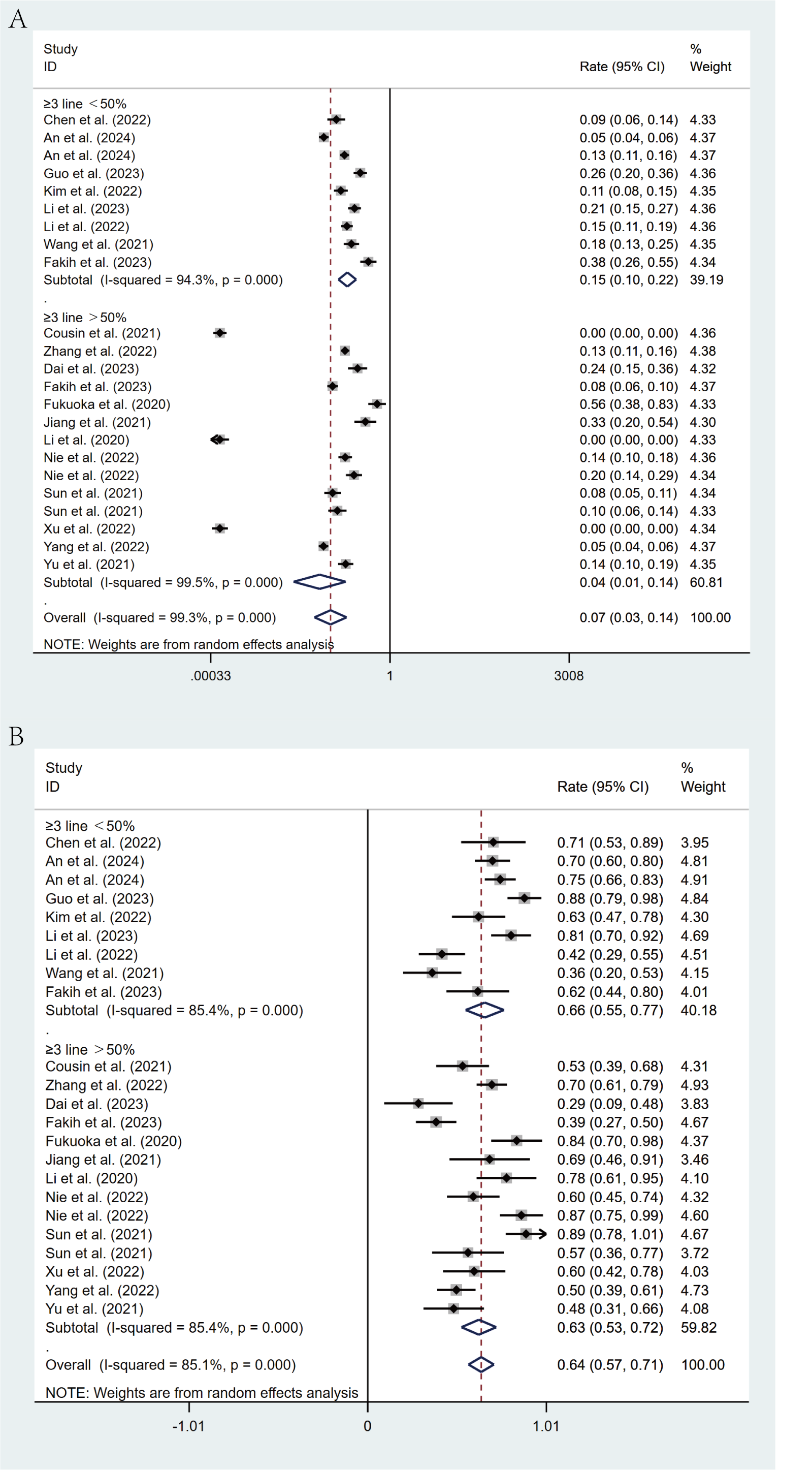

Supplement: Supplementary Figure 4 — Forest plot of the subgroup analysis for ORR (A) and DCR (B) across different lines of treatment (≥ third-line <50% vs. ≥ third-line >50%). [file Image4.tif]

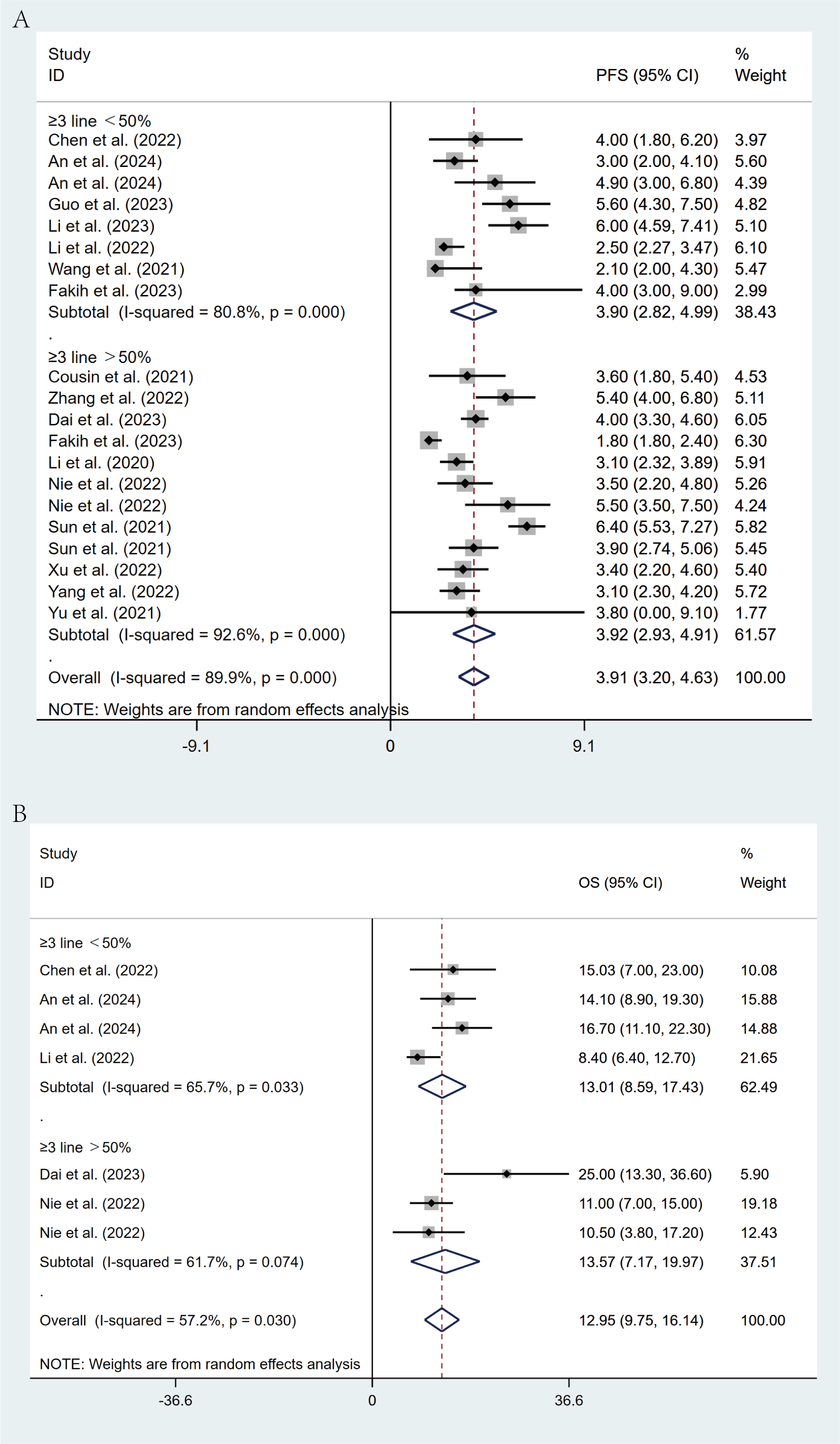

Supplement: Supplementary Figure 5 — Forest plot of the subgroup analysis for PFS (A) and OS (B) across different lines of treatment (≥ third-line <50% vs. ≥ third-line >50%). [file Image5.tif]

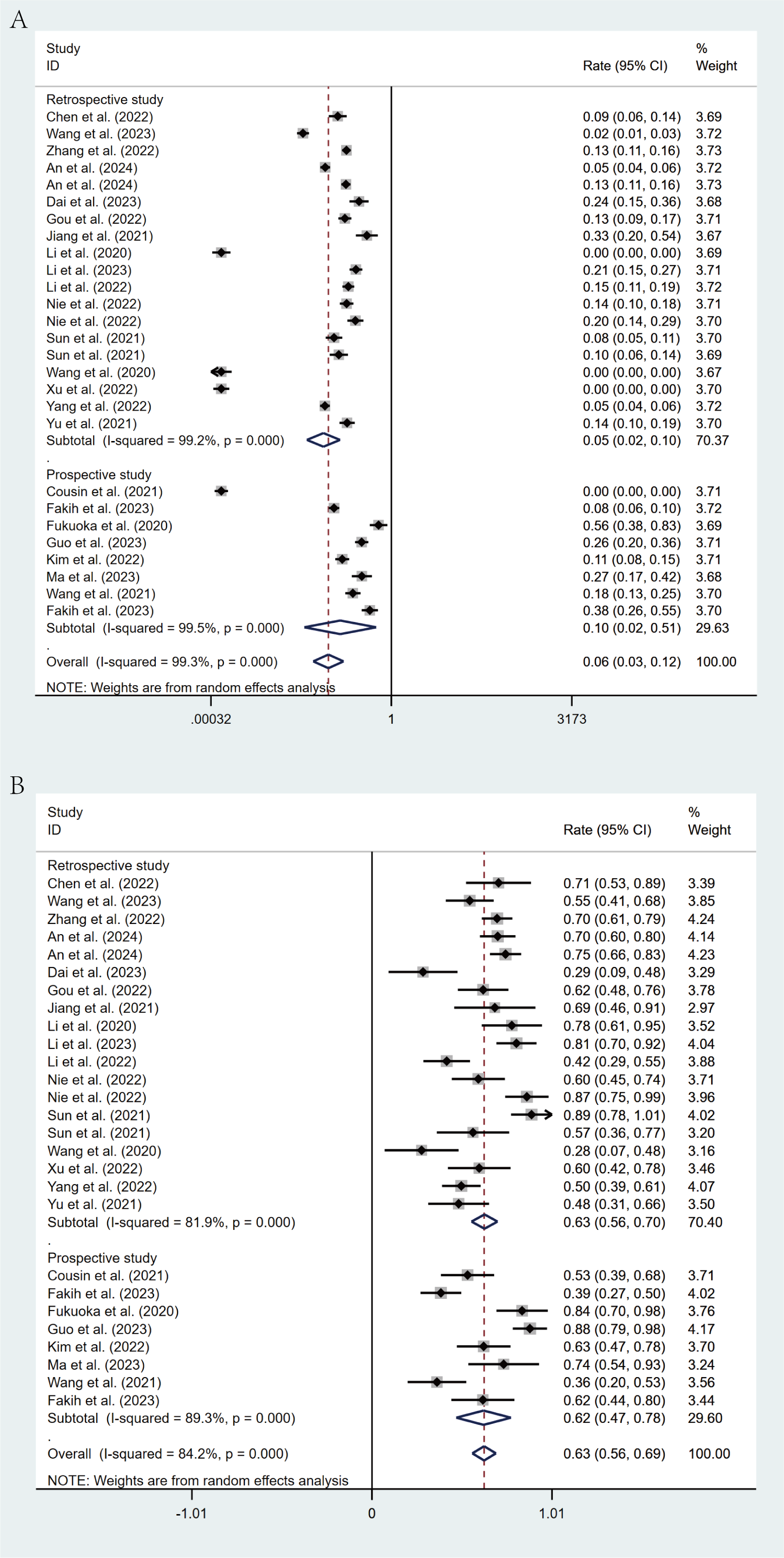

Supplement: Supplementary Figure 6 — Forest plot of the subgroup analysis for ORR (A) and DCR (B) across different study designs (Retrospective study vs. Prospective study). [file Image6.tif]

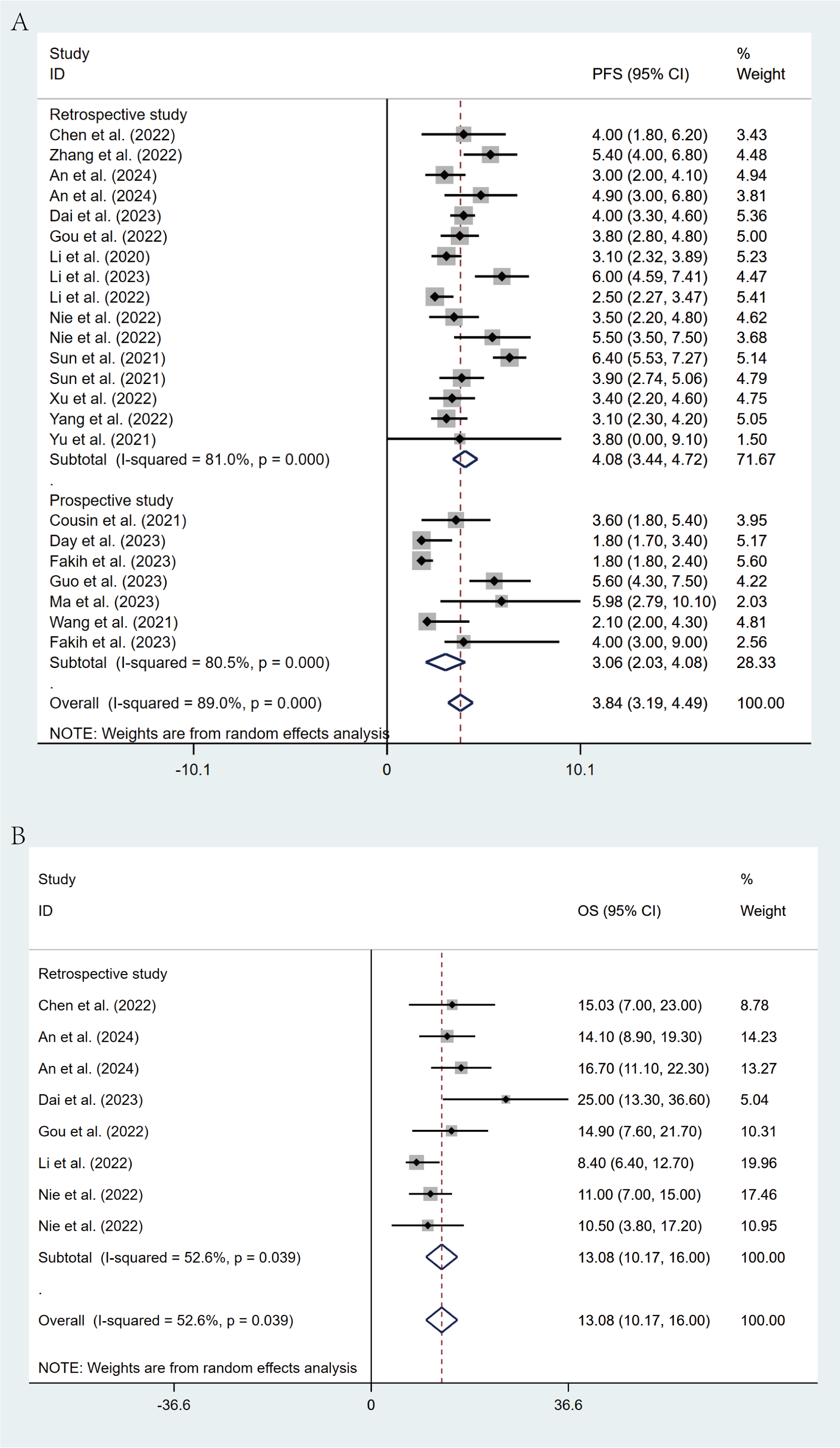

Supplement: Supplementary Figure 7 — Forest plot of the subgroup analysis for PFS (A) and OS (B) across different study designs (Retrospective study vs. Prospective study). [file Image7.tif]

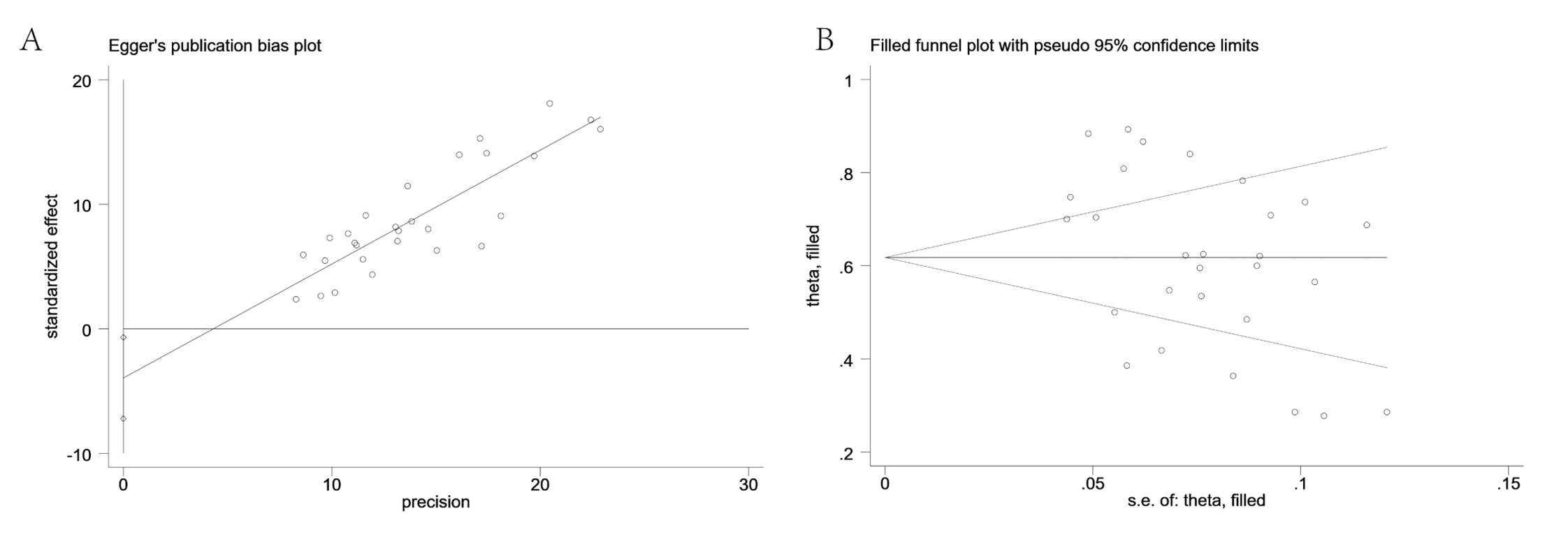

Supplement: Supplementary Figure 8 — Publication bias assessment for disease control rate (DCR) using Egger’s test (A) and trim-and-fill method (B). [file Image8.tif]
