# Supplementary material for: The efficacy and safety of regorafenib/fruquintinib combined with PD-1/PD-L1 for metastatic colorectal cancer: a meta-analysis based on single-arm studies
Source: Front Immunol. 2025 May 29;16:1579293. doi: 10.3389/fimmu.2025.1579293 (PMC12159013; doi:10.3389/fimmu.2025.1579293)
Supplement: Supplementary file 9 [file Table1.docx]

Table S1 Search Result Record

| Pubmed | | |
| --- | --- | --- |
| # | Query | Results |
| 1 | Colorectal Neoplasms[MeSH Terms] | 247,571 |
| 2 | "colo rectal metastases"[Title/Abstract] OR "Colorectal Cancer*"[Title/Abstract] OR "Colorectal Carcinoma*"[Title/Abstract] OR "colorectal metastas*s"[Title/Abstract] OR "Colorectal Neoplasm*"[Title/Abstract] OR "Colorectal Tumor*"[Title/Abstract] OR "metastatic colo rectal"[Title/Abstract] OR "metastatic colorectal"[Title/Abstract] | 162,657 |
| 3 | #1 OR #2 | 295,891 |
| 4 | "regorafenib" [Supplementary Concept] | 935 |
| 5 | "bay*73*4506"[Title/Abstract] OR "regorafenib"[Title/Abstract] OR "resihance"[Title/Abstract] OR "ro*7069680"[Title/Abstract] OR "stivarga"[Title/Abstract] | 2,062 |
| 6 | "HMPL-013" [Supplementary Concept] | 51 |
| 7 | "elunate"[Title/Abstract] OR "fruquintinib"[Title/Abstract] OR "fruzaqla"[Title/Abstract] OR "hmpl*013"[Title/Abstract] OR "tak*113"[Title/Abstract] | 133 |
| 8 | #4 OR #5 OR #6 OR #7 | 2,208 |
| 9 | Immune Checkpoint Inhibitors[MeSH Terms] | 11,392 |
| 10 | "22C3 pharmDx"[Title/Abstract] OR "28 8 pharmDx"[Title/Abstract] OR "CTLA 4 Inhibitor*"[Title/Abstract] OR "Cytotoxic T Lymphocyte Associated Protein 4 Inhibitor*"[Title/Abstract] OR "enzyme immunohistochemistry IVD kit programmed death ligand 1 PD L1 "[Title/Abstract] OR "Immune Checkpoint Block*"[Title/Abstract] OR "Immune Checkpoint Inhibitor*"[Title/Abstract] OR "PD 1 PD L1 Blockade"[Title/Abstract] OR "PD 1 Inhibitor*"[Title/Abstract] OR "PD L1 22C3 pharmDx"[Title/Abstract] OR "PD L1 assay kit"[Title/Abstract] OR "PD L1 detection kit"[Title/Abstract] OR "PD L1 test kit"[Title/Abstract] OR "PD L1 Inhibitor*"[Title/Abstract] OR "Programmed Cell Death Protein 1 Inhibitor*"[Title/Abstract] OR "Programmed Death Ligand 1 immunohistochemistry assay"[Title/Abstract] OR "Programmed Death Ligand 1 Inhibitors"[Title/Abstract] OR "Programmed Death Ligand 1 test kit"[Title/Abstract] OR "Ventana PD L1"[Title/Abstract] OR "nivolumab"[Title/Abstract] OR "pembrolizumab"[Title/Abstract] OR "atezolizumab"[Title/Abstract] OR "durvalumab"[Title/Abstract] OR "avelumab"[Title/Abstract] OR "nofazinlimab"[Title/Abstract] OR "Sintilimab"[Title/Abstract] OR "Camrelizumab"[Title/Abstract] OR "Tislelizumab"[Title/Abstract] OR "Toripalimab"[Title/Abstract] | 51,927 |
| 11 | #9 OR #10 | 53,554 |
| 12 | #3 AND #8 AND #11 | 83 |

| Embase | | |
| --- | --- | --- |
| # | Query | Results |
| 1 | 'metastatic colorectal cancer'/exp | 26339 |
| 2 | 'colo rectal metastases':ab,ti,kw OR 'colorectal cancer*':ab,ti,kw OR 'colorectal carcinoma*':ab,ti,kw OR 'colorectal metastas*s':ab,ti,kw OR 'colorectal neoplasm*':ab,ti,kw OR 'colorectal tumor*':ab,ti,kw OR 'metastatic colo rectal':ab,ti,kw OR 'metastatic colorectal':ab,ti,kw | 243060 |
| 3 | 'regorafenib'/exp | 7792 |
| 4 | 'bay*73*4506':ab,ti,kw OR 'regorafenib':ab,ti,kw OR 'resihance':ab,ti,kw OR 'ro*7069680':ab,ti,kw OR 'stivarga':ab,ti,kw | 4045 |
| 5 | 'fruquintinib'/exp | 384 |
| 6 | 'elunate':ab,ti,kw OR 'fruquintinib':ab,ti,kw OR 'fruzaqla':ab,ti,kw OR 'hmpl*013':ab,ti,kw OR 'tak*113':ab,ti,kw | 246 |
| 7 | 'pd-l1 test kit'/exp | 739 |
| 8 | '22c3 pharmdx':ab,ti,kw OR '28 8 pharmdx':ab,ti,kw OR 'ctla 4 inhibitor*':ab,ti,kw OR 'cytotoxic t lymphocyte associated protein 4 inhibitor*':ab,ti,kw OR 'enzyme immunohistochemistry ivd kit programmed death ligand 1 pd l1':ab,ti,kw OR 'immune checkpoint block*':ab,ti,kw OR 'immune checkpoint inhibitor*':ab,ti,kw OR 'pd 1 pd l1 blockade':ab,ti,kw OR 'pd 1 inhibitor*':ab,ti,kw OR 'pd l1 22c3 pharmdx':ab,ti,kw OR 'pd l1 assay kit':ab,ti,kw OR 'pd l1 detection kit':ab,ti,kw OR 'pd l1 test kit':ab,ti,kw OR 'pd l1 inhibitor*':ab,ti,kw OR 'programmed cell death protein 1 inhibitor*':ab,ti,kw OR 'programmed death ligand 1 immunohistochemistry assay':ab,ti,kw OR 'programmed death ligand 1 inhibitors':ab,ti,kw OR 'programmed death ligand 1 test kit':ab,ti,kw OR 'ventana pd l1':ab,ti,kw OR 'nivolumab':ab,ti,kw OR 'pembrolizumab':ab,ti,kw OR 'atezolizumab':ab,ti,kw OR 'durvalumab':ab,ti,kw OR 'avelumab':ab,ti,kw OR 'nofazinlimab':ab,ti,kw OR 'sintilimab':ab,ti,kw OR 'camrelizumab':ab,ti,kw OR 'tislelizumab':ab,ti,kw OR 'toripalimab':ab,ti,kw | 91165 |
| 9 | (#1 OR #2) AND (#3 OR #4 OR #5 OR #6) AND (#7 OR #8) | 257 |

| Cochrane Library | | |
| --- | --- | --- |
| # | Query |  |
| 1 | MeSH descriptor: [Colorectal Neoplasms] explode all trees | 12759 |
| 2 | ('colo rectal metastases' OR 'Colorectal Cancer*' OR 'Colorectal Carcinoma*' OR 'colorectal metastas*s' OR 'Colorectal Neoplasm*' OR 'Colorectal Tumor*' OR 'metastatic colo rectal' OR 'metastatic colorectal'):ab,ti,kw | 20993 |
| 3 | ('bay*73*4506' OR 'regorafenib' OR 'resihance' OR 'ro*7069680' OR 'stivarga'):ab,ti,kw | 709 |
| 4 | ('elunate' OR 'fruquintinib' OR 'fruzaqla' OR 'hmpl*013' OR 'tak*113'):ab,ti,kw | 84 |
| 5 | MeSH descriptor: [Immune Checkpoint Inhibitors] explode all trees | 275 |
| 6 | ('22C3 pharmDx' OR '28 8 pharmDx' OR 'CTLA 4 Inhibitor*' OR 'Cytotoxic T Lymphocyte Associated Protein 4 Inhibitor*' OR 'enzyme immunohistochemistry IVD kit programmed death ligand 1 PD L1 ' OR 'Immune Checkpoint Block*' OR 'Immune Checkpoint Inhibitor*' OR 'PD 1 PD L1 Blockade' OR 'PD 1 Inhibitor*' OR 'PD L1 22C3 pharmDx' OR 'PD L1 assay kit' OR 'PD L1 detection kit' OR 'PD L1 test kit' OR 'PD L1 Inhibitor*' OR 'Programmed Cell Death Protein 1 Inhibitor*' OR 'Programmed Death Ligand 1 immunohistochemistry assay' OR 'Programmed Death Ligand 1 Inhibitors' OR 'Programmed Death Ligand 1 test kit' OR 'Ventana PD L1' OR 'nivolumab' OR 'pembrolizumab' OR 'atezolizumab' OR 'durvalumab' OR 'avelumab' OR 'nofazinlimab' OR 'Sintilimab' OR 'Camrelizumab' OR 'Tislelizumab' OR 'Toripalimab'):ab,ti,kw | 16074 |
| 7 | (#1 OR #2) AND (#3 OR #4) AND (#5 OR #6) | 61 |

| Web of Science | | |
| --- | --- | --- |
| # | Query | Results |
| 1 | (TS=((colo rectal metastases) OR (Colorectal Cancer*) OR (Colorectal Carcinoma*) OR (colorectal metastas*s) OR (Colorectal Neoplasm*) OR (Colorectal Tumor*) OR (metastatic colo rectal) OR (metastatic colorectal))) NOT (SILOID==("PPRN")) | 427785 |
| 2 | TS=((bay*73*4506) OR (regorafenib) OR (resihance) OR (ro*7069680) OR (stivarga)) and Preprint Citation Index (Exclude – Database) | 3967 |
| 3 | TS=((elunate) OR (fruquintinib) OR (fruzaqla) OR (hmpl*013) OR (tak*113)) and Preprint Citation Index (Exclude – Database) | 252 |
| 4 | TS=((22C3 pharmDx) OR (28 8 pharmDx) OR (CTLA 4 Inhibitor*) OR (Cytotoxic T Lymphocyte Associated Protein 4 Inhibitor*) OR (enzyme immunohistochemistry IVD kit programmed death ligand 1 PD L1 ) OR (Immune Checkpoint Block*) OR (Immune Checkpoint Inhibitor*) OR (PD 1 PD L1 Blockade) OR (PD 1 Inhibitor*) OR (PD L1 22C3 pharmDx) OR (PD L1 assay kit) OR (PD L1 detection kit) OR (PD L1 test kit) OR (PD L1 Inhibitor*) OR (Programmed Cell Death Protein 1 Inhibitor*) OR (Programmed Death Ligand 1 immunohistochemistry assay) OR (Programmed Death Ligand 1 Inhibitors) OR (Programmed Death Ligand 1 test kit) OR (Ventana PD L1) OR (nivolumab) OR (pembrolizumab) OR (atezolizumab) OR (durvalumab) OR (avelumab) OR (nofazinlimab) OR (Sintilimab) OR (Camrelizumab) OR (Tislelizumab) OR (Toripalimab)) and Preprint Citation Index (Exclude – Database) | 159730 |
| 5 | #1 AND (#2 OR #3 ) AND #4 and Preprint Citation Index (Exclude – Database) | 201 |
