# Supplementary material for: The efficacy and safety of regorafenib/fruquintinib combined with PD-1/PD-L1 for metastatic colorectal cancer: a meta-analysis based on single-arm studies
Source: Front Immunol. 2025 May 29;16:1579293. doi: 10.3389/fimmu.2025.1579293 (PMC12159013; doi:10.3389/fimmu.2025.1579293)
Supplement: Supplementary file 10 [file Table2.docx]

Table S2 Medication Information and AEs Leading to drug interruption/dose reduction and serious irAEs in this Study

| **Study,**  **year** | **Intervention** | **Drug initial dose** | **Categories of ICs** | **Dose interruption or reduction ralated AEs** | **Serious irAEs** |
| --- | --- | --- | --- | --- | --- |
| Chen et al., 2022(39) | Regorafenib | Regorafenib: 120mg(16.7%) 80mg(79.2%） 40mg(4.2%) | Sintilimab(50.0%) Carrelizumab(25.0%) Nivolumab(8.3%) Toripalimab(8.3%) Pembrolizumab(4.2%) Tislelizumab(4.2%) | Dose reduction： hand-foot skin reaction（12.5%） hypertension（4.2%） proteinuria（4.2%） | NR |
| Cousin et al., 2021(27) | Regorafenib | Regorafenib: 160 mg(100%) | Avelumab(100%) | NR | NR |
| Wang et al., 2023(40) | Regorafenib | Regorafenib: 160 mg(29%) 120 mg(17%) 80mg(48%) 40mg(6%) | NR | NR | NR |
| Zhang et al., 2022(30) | Fruquintinib | Regorafenib: 5mg(92.7%) 4mg(0.9%) 3mg(6.4%) | Sintilimab(46.4%) Camrelizumab(35.5%) Toripalimab(13.6%) Tislelizumab(3.6%) Pembrolizumab(0.9%) | Fruquintinib dose reduction:hand-foot syndromez(1.8%) ,hypertension(0.9%) Dose discontinued:proteinuria (1.8%), rash (1.8%), hypertension(0.9%), hand-foot syndrome(0.9%),oral mucositis(0.9%), thrombocytopenia(0.9%), diarrhea(0.9%), arrhythmia(0.9%), adrenal cortex hypofunction(0.9%), immune-associated pneumonia(0.9%). | adrenal cortex hypofunction(0.9%), pneumonia(0.9%). |
| An et al., 2024(41) | Regorafenib | Regorafenib: 80mg (44.4%) 120mg (38.3%)  160mg (14.8%)  Unknown (2.5%) | Camrelizumab(65.5%） Sintilimab(22.2%) Tislelizumab(1.2%) Nivolumab(8.6%) Toripalimab(2.5%) | serious AE:gastrointestinal hemorrhage(1.2%),heart failure(1.2%) | NR |
|  | Fruquintinib | Fruquintinib: 3mg (8.4%)  4mg (2.1%) 5mg(89.5%) | Sintilimab(47.4%) Camrelizumab(29.5%) Toripalimab(10.5%) Tislelizumab(9.5%) Pembrolizumab(2.1%) Nivolumab(1.0%) | serious AE:heart failure(1.1%),rectovaginal fistulae（1.1%） | NR |
| Dai et al., 2023(29) | Regorafenib | Regorafenib: 80mg-120mg | Nivolumab(61.9%) Toripalimab(19%) Serplulimab(9.5%) Sintilimab(9.5%) | NR | NR |
| Day et al., 2023(42) | Regorafenib | Regorafenib: 80mg(50%) 120mg (50%) | Nofazinlimab | Dose interruption:pyrexia(7.1%) | DLTs grade 3 irAE: colitis and maculo-papular rash(7.1%) Grade 3 irAE：Pyrexia(7.1%)、Rash(7.1%)、Maculo-papular rash(14.2%)、Colitis(7.1%)、Lymphocyte count decreased(7.1%)、Seizure(7.1%)、Thrombocytopenia(7.1%) |
| Fakih et al., 2023(43) | Regorafenib | Regorafenib: 80 mg | Nivolumab | NR | irAE in 24 patients (34%) and were serious in six patients (9%). The most frequent AEs :skin toxicities (16%), maculopapular rash (7%), rash(3%), pruritis(3%), exfoliation(3%) ,immune/treatment-related diarrhoea (6%) |
| Fukuoka et al., 2020(14) | Regorafenib | Regorafenib: 80mg(56%) 120mg(40%) 160mg(4%) | Nivolumab | Dose reduction :palmar plantar erythrodysesthesia or maculopapular rash | NR |
| Gou et al., 2022(44) | Fruquintinib | Fruquintinib: 5 mg and 3 mg | sintilimab(60.0%) camrelizumab(17.8%) pembrolizumab(15.6%) nivolumab(6.7%) | NR | NR |
| Guo et al., 2023(45) | Fruquintinib | Fruquintinib:  5 mg 2w/1w regimen（50%） Fruquintinib: 3 mg continuous regimen（50%） | sintilimab | TRAEs leading to interruption of fruquintinib and sintilimab were reported in two (4.5%) and three (6.8%)patients, respectively | Immune-related adverse events related to sintilimab were observed in 32 (72.7%) patients. |
| Jiang et al., 2021(15) | Regorafenib/  Fruquintinib | Regorafenib：80 mg fruquintinib：3 mg | Camrelizumab | NR | Reactive cutaneous capillary  endothelial proliferation(6.3%) |
| Kim et al., 2022(28) | Regorafenib | Regorafenib 120 mg (9.8%) Regorafenib 80 mg (90.2%) | nivolumab | seven interruption treatment due to adverse events | Grade 3 or 4 immune-mediated AEs observed in eight patients, including rash (9.6%) |
| Li et al., 2020(46) | Regorafenib | regorafenib 80mg（47.8%） regorafenib 120mg（13.0%） regorafenib 160 mg（39.1%） | nivolumab camrelizumab toripalimab pembrolizumab | regorafenib interruption:grade 3 hoarseness(4.3%) 、grade 3 palmar-plantar erythrodysesthesia(8.7%), rash (4.3%)、liver dysfunction（4.3%） | NR |
| Li et al., 2023(47) | Fruquintinib | Fruquintinib: 4 mg | sintilimab | Dose interruption:severe rash（2.1%） | NR |
| Li et al., 2022(48) | Regorafenib | NR | Sintilimab (64.0%) Nivolumab(9.7%) Toripalimab (17.5%) Camrelizumab(7.8%) Pembrolizumab (1.0%) | NR | NR |
| Ma et al., 2023(49) | Fruquintinib | Fruquintinib: 5 mg | Toripalimab | Fruquintinib dose interruption:Hypertension(15.79%) Toripalimab dose interruption:Hepatic dysfunction (15.79%) | Hepatic dysfunction (15.79%) |
| Nie et al., 2022(50) | Regorafenib | Regorafenib: 80 mg | sintilimab | The dose reduction:28 (38.9%) and treatment interruptions 33 (45.8%). The most common treatment-related hematological AEs: increased ALT/AST (18.1%), anemia (15.3%), decreased white blood count (8.3%), hyperbilirubinemia (6.9%), and decreased platelet (6%). | NR |
|  | Fruquintinib | Fruquintinib: 5 mg | sintilimab | NR | NR |
| Qu et al.,2024(51) | Regorafenib | Regorafenib: 80 mg (55.7%) 120 mg (24.6%) 160mg(18.9%) | Sintilimab(31.0%) Toripalimab(25.5%) Camrelizumab(24.1%) Tieslelizumab(4.8%) Pembrolizumab(4.1%) | NR | NR |
| Sun et al., 2021(52) | Fruquintinib | Fruquintinib: 3 mg (71.4%) 4 mg (17.9%) 5 mg (10.7%) | sintilimab (53.6%) camrelizumab (46.4%) | NR | one patient died of immune myocarditis |
|  | Regorafenib | Regorafenib: 80 mg(73.9%) 120 mg(21.7%) 160 mg(4.3%) | camrelizumab (52.2%) | No | NR |
| Wang et al., 2020(53) | Regorafenib | Regorafenib: 80 mg | nivolumab(94.4%) pembrolizumab(5.6%) | Dose interruptionTRAEs: headache(7.1%) , impaired liver function (7.1%),infectious pneumonia (2.4%), hand foot syndrome (2.4%), rash (2.4%), and frequent premature ventricular contractions (2.4%). | irAE Grade 3: rash (5.1%), impaired liver function (5.1%), and diarrhea (2.5%), |
| Wang et al., 2021(54) | Regorafenib | Regorafenib: 80 mg (92.9%) 120mg(7.1%) | toripalimab | Dose reduction:hand-foot syndrome(6.7%), abnormal capillary proliferation(3.3%) Discontinuation:transamiNRse elevation(3.3%) | NR |
| Xu et al., 2022(55) | Regorafenib | Regorafenib: 80 mg(18) 120 mg(8) 160 mg(4) | sintilimab(30%) toripalimab(26.7%) camrelizumab(16.7%) tislelizumab(13.3%) nivolumab(10%) pembrolizumab(3.3%) | NR | NR |
| Yang et al., 2022(56) | Regorafenib | Regorafenib: 80 mg(76%) | sintilimab (39%) nivolumab (20%) toripalimab (15%) camrelizumab (14%) pembrolizumab (7%) tislelizumab(4%) | NR | NR |
| Yu et al.,2021(57) | Regorafenib | Regorafenib: 80mg or 120mg | toripalimab | regorafenib dose interruption:skin toxic effects(31%) irAEs dose interruptions: hepatitis(13.8%), colitis(6.9%),  meningitis(3.4%), squamous cell carcinoma of the skin(3.4%) | irAEs grade 3 or higher: rash (37.9%), elevation of aspartate aminotransferase or alanine aminotransferase level (13.8%), myalgia (10.3%) |
| Fakih et al.,2023(58) | Regorafenib | Regorafenib: 80mg | nivolumab | Dose reduction： hand-foot skin reaction（12.5%） hypertension（4.2%） proteinuria（4.2%） | NR |

NR, not reported;;ICs,immune checkpoints;AEs,adverse events;irAEs,immune-related adverse events.
