# Supplementary material for: The efficacy and safety of regorafenib/fruquintinib combined with PD-1/PD-L1 for metastatic colorectal cancer: a meta-analysis based on single-arm studies
Source: Front Immunol. 2025 May 29;16:1579293. doi: 10.3389/fimmu.2025.1579293 (PMC12159013; doi:10.3389/fimmu.2025.1579293)
Supplement: Supplementary file 11 [file Table3.docx]

Table S3 Quality assessment of the studies included in the meta-analysis

| Study | Q1 | Q2 | Q3 | Q4 | Q5 | Q6 | Q7 | Q8 | Quality score |
| --- | --- | --- | --- | --- | --- | --- | --- | --- | --- |
| Chen et al., 2022 | 2 | 2 | 0 | 2 | 1 | 2 | 1 | 0 | 10 |
| Cousin et al., 2021 | 2 | 2 | 2 | 2 | 1 | 2 | 1 | 2 | 14 |
| Wang et al., 2023 | 2 | 2 | 0 | 2 | 1 | 2 | 1 | 0 | 10 |
| Zhang et al., 2022 | 2 | 2 | 0 | 2 | 1 | 2 | 1 | 0 | 10 |
| An et al., 2024 | 2 | 2 | 0 | 2 | 1 | 2 | 1 | 0 | 10 |
| Dai et al., 2023 | 2 | 2 | 0 | 2 | 1 | 2 | 1 | 0 | 10 |
| Day et al., 2023 | 2 | 2 | 2 | 2 | 1 | 2 | 1 | 2 | 14 |
| Fakih et al., 2023 | 2 | 2 | 2 | 2 | 1 | 2 | 1 | 2 | 14 |
| Fukuoka et al., 2020 | 2 | 2 | 2 | 2 | 2 | 2 | 2 | 2 | 16 |
| Gou et al., 2022 | 2 | 2 | 0 | 2 | 2 | 2 | 2 | 0 | 12 |
| Guo et al., 2023 | 2 | 2 | 2 | 2 | 1 | 2 | 2 | 2 | 15 |
| Jiang et al., 2021 | 2 | 2 | 0 | 2 | 1 | 2 | 1 | 0 | 10 |
| Kim et al., 2022 | 2 | 2 | 2 | 2 | 1 | 2 | 2 | 2 | 15 |
| Li et al., 2020 | 2 | 2 | 0 | 2 | 1 | 2 | 1 | 0 | 10 |
| Li et al., 2023 | 2 | 2 | 0 | 2 | 1 | 2 | 1 | 0 | 10 |
| Li et al., 2022 | 2 | 2 | 0 | 2 | 1 | 2 | 1 | 0 | 10 |
| Ma et al., 2023 | 2 | 2 | 2 | 2 | 1 | 2 | 2 | 0 | 13 |
| Nie et al., 2022 | 2 | 2 | 2 | 2 | 1 | 2 | 2 | 0 | 13 |
| Qu et al.,2024 | 2 | 2 | 2 | 2 | 1 | 2 | 2 | 0 | 13 |
| Sun et al., 2021 | 2 | 2 | 0 | 2 | 1 | 2 | 2 | 0 | 11 |
| Wang et al., 2020 | 2 | 2 | 0 | 2 | 1 | 2 | 2 | 0 | 11 |
| Wang et al., 2021 | 2 | 2 | 2 | 2 | 1 | 2 | 2 | 2 | 15 |
| Xu et al., 2022 | 2 | 2 | 0 | 2 | 1 | 2 | 2 | 0 | 11 |
| Yang et al., 2022 | 2 | 2 | 0 | 2 | 1 | 2 | 2 | 0 | 11 |
| Yu et al.,2021 | 2 | 2 | 2 | 2 | 1 | 2 | 2 | 2 | 15 |
| Fakih et al.,2023 | 2 | 2 | 2 | 2 | 1 | 2 | 2 | 2 | 15 |

Numbers Q1-Q8 in heading signified: Q1, **A clearly stated aim**: The question addressed should be precise and relevant in the light of available literature; Q2, **Inclusion of consecutive patients:**All patients potentially fit for inclusion (satisfying the criteria for inclusion) have been included in the study during the study period (no exclusion or details about the reasons for exclusion); Q3, **Prospective collection of data:**Data were collected according to a protocol established before the beginning of the study; Q4, **Endpoints appropriate to the aim of the study:** Unambiguous explanation of the criteria used to evaluate the main outcome which should be in accordance with the question addressed by the study. Also, the endpoints should be assessed on an intention-to-treat basis; Q5, **Unbiased assessment of the study endpoint**:Blind evaluation of objective endpoints and double-blind evaluation of subjective endpoints. Otherwise the reasons for not blinding should be stated; Q6, **Follow-up period appropriate to the aim of the study**:The follow-up should be sufficiently long to allow the assessment of the main endpoint and possible adverse events; Q7, **Loss to follow up less than 5%:**All patients should be included in the follow up. Otherwise, the proportion lost to follow up should not exceed the proportion experiencing the major endpoint; Q8, **Prospective calculation of the study size**:Information of the size of detectable difference of interest with a calculation of 95% confidence interval, according to the expected incidence of the outcome event, and information about the level for statistical significance and estimates of power when comparing the outcomes.
