# Supplementary material for: The efficacy and safety of regorafenib/fruquintinib combined with PD-1/PD-L1 for metastatic colorectal cancer: a meta-analysis based on single-arm studies
Source: Front Immunol. 2025 May 29;16:1579293. doi: 10.3389/fimmu.2025.1579293 (PMC12159013; doi:10.3389/fimmu.2025.1579293)
Supplement: Supplementary file 12 [file Table4.docx]

Table S4 Potential biomarkers in this study

| **Study, year** | **Potential biomarkers** |
| --- | --- |
| Chen et al., 2022(39) | NR |
| Cousin et al., 2021(27) | CD8 high versus low: mPFS-3.7 mo versus 2.3 mo (P=0.035) TAM2 high versus low:mPFS-1.8 mo versus 3.7 mo (P=0.002) PD-L1 TPS≥10 versus <10:no statistical difference in PFS TMB high versus low: no statistical difference in PFS |
| Wang et al., 2023(40) | NR |
| Zhang et al., 2022(30) | Liver versus non-liver metastasis:mPFS-3.4 mo vs 7.6 mo(P＜0.05) ALP>160 U/L vs ALP≤160 U/L:mPFS-3.4 mo vs 7.6 mo(P＜0.05) FIB>4g/L vs FIB≤4g/L:mPFS-3.4 mo vs 6.8 mo(P＜0.05) D-dimer >0.3 mg vs D-dimer ≤0.3 mg:mPFS-4.1 mo vs 6.3 mo(P＜0.05) ALP decreased vs ALP increased:mPFS-7.0 mo vs 3.4 mo(P＜0.05) TMB-L vs TMB-H:mPFS-5.0 mo vs. 6.1 mo(P=0.979) six patients BRAF mutations:PR(33.3%), SD(66.7%),ORR (33.3%), DCR (83.3%), PFS:13.3 mo |
| An et al., 2024(41) | NR |
|  | BRAF mutation:survival benefits |
| Dai et al., 2023(29) | ERBB2/3 mutation vs ERBB2/3 WT:mPFS-15 mo vs 4 mo(p = 0.01), OS-25 mo vs 12mo(p = 0.238) |
| Day et al., 2023(42) | NR |
| Fakih et al., 2023(43) | Liver versus non-liver metastasis: mPFS-1.9 mo versus 3.5 mo (P = unknown),ORR-0 versus 22% (P= unknown) |
| Fukuoka et al., 2020(14) | PD-L1 CPS≥1 versus<1: mPFS 6 mo versus NR (P=unknown) TMB high versus low:mPFS -12.5 mo versus 7.9 mo (P = unknown) |
| Gou et al., 2022(44) | NR |
| Guo et al., 2023(45) | PD-L1 CPS＜1 vs PD-L1 CPS≥1:mPFS clinical benefits  PD-L1 CPS＜1(N=23) vs PD-L1 CPS≥1(N=4):mOS-17.3 mo vs 9.9 mo(results should be interpreted with caution) |
| Jiang et al., 2021(15) | NR |
| Kim et al., 2022(28) | Liver versus non-liver metastasis: mPFS-2.3 mo versus 8.9 mo (P= 0.02) Lung versus non-lung metastasis: mPFS-5.6 mo versus 2.3 mo (P=0.02) Treg low versus high:mPFS-9.8 mo versus 1.9 mo (P=0.01) PD-L1 TPS ≥1 versus<1: mPFS-2.8 mo versus 5.6 mo (P=0.44) CD8 high versus low:mPFS-9.8 mo versus 3.7 mo (P=0.15) |
| Li et al., 2020(46) | Liver versus non-liver metastasis: mPFS-2.3 mo versus 3.5 mo (p = 0.34) KRAS mutation versus wild type: no statistical difference in mPFS(p = 0.69) |
| Li et al., 2023(47) | No statistically significant predictive factors were found |
| Li et al., 2022(48) | NR |
| Ma et al., 2023(49) | RAS gene status showed no PFS benefit |
| Nie et al., 2022(50) | Liver versus non-liver metastasis:ORR: 9.1% vs. 21.4%(P = unknown), mOS： 10.0 vs. 26.0 mo,（P=0.016） |
|  | KRAS mutation versus wild type:mOS-13.0 mo vs. 9.2 mo(HR=0.56), |
| Qu et al.,2024(51) | NR |
| Sun et al., 2021(52) | NR |
|  | TMB was available in 15 patients, with low TMB observed among all patients. The relationship between TMB and efficacy was not analyzed. |
| Wang et al., 2020(53) | BRAF mutation(2),ORR=50% Fusobacterium of gut microbiome low vs high:mPFS-5.2 mo vs 2.0 mo (P=0.002) |
| Wang et al., 2021(54) | low-PLR vs high-PLR:mPFS-4.2 mo vs 2.8 mo (P = 0.005) |
| Xu et al., 2022(55) | NLR ≥ 1.5: shorter PFS (HR 3.43; 95%CI 1.24–9.54, P = 0.02) |
| Yang et al., 2022(56) | NR |
| Yu et al.,2021(57) | 6 patients with RAS/BRAF wild-type variations showed no response 3 patients BRAF mutation showed response all patients with available results had a low TMB ctDNR decreased by 50% :mPFS-9.5 months (range, 3-23 months) |
| Fakih et al.,2023(58) | NR |

NR, not reported;;ICs,immune checkpoints;AEs,adverse events;irAEs,immune-related adverse events.
